# Cretaceous amber fossils highlight the evolutionary history and morphological conservatism of land snails

**DOI:** 10.1038/s41598-019-51840-3

**Published:** 2019-11-04

**Authors:** Takahiro Hirano, Kaito Asato, Shûhei Yamamoto, Yui Takahashi, Satoshi Chiba

**Affiliations:** 1 Department of Biological Sciences, University of Idaho, Moscow, USA; 2 Faculty of Life and Environmental Sciences, University of Tsukuba, Ibaraki, Japan; 3 Integrative Research Center, Field Museum of Natural History, Chicago, USA; 4 Muroto Geopark Promotion Committee, Muroto Global Geopark Center, Kochi, Japan; 5 Center for Northeast Asian Studies, Tohoku University, Miyagi, Japan; 6 Graduate school of Life Sciences, Tohoku University, Miyagi, Japan

**Keywords:** Phylogenetics, Palaeontology

## Abstract

Other than hard bones and shells, it is rare for soft tissues to fossilize, but occasionally they are well-preserved in amber. Here, we focus on both modern and fossilized species of the land snail superfamily Cyclophoroidea. Phylogenetic relationships within the Cyclophoroidea were previously studied using extant species, but timing of divergence within the group remains unclear. In addition, it is difficult to observe morphological traits such as the chitinous operculum and periostracum of fossil snails due to their poor preservation potential. Here we describe nine species including a new genus and five new species of well-preserved fossil cyclophoroideans from the mid-Cretaceous Burmese amber. These fossils include not only the shell, but also the chitinous operculum and periostracum, soft body, and excrements. We present the first estimation of divergence time among cyclophoroidean families using fossil records and molecular data, suggesting extreme morphological conservatism of the Cyclophoroidea for nearly 100 million years.

## Introduction

Understanding how phenotypic divergence occurs is a central subject of evolutionary biology and paleontology. Molecular-based approaches can clarify phylogenetic relationships and evolutionary patterns of phenotypic divergence^1^, whereas fossils can be used to study how traits of ancient organisms differ from present forms^2^. However, the majority of fossils only provide insight on the morphology of structures that can withstand the fossilization process, severely limiting our knowledge of phenotypic evolution of soft tissues and organs. Mid-Cretaceous Burmese amber from northern Myanmar has attracted considerable attention from both scientists and the public in recent years, because it contains many exceptionally preserved terrestrial organisms (e.g. a dinosaur tail and birds^3,4^). Fossils like these can improve our understanding of trait evolution and provide reliable calibration points for dating phylogenies. While combining molecular phylogeny and paleontological studies can help to understand evolutionary patterns of phenotypic divergence^5^, few studies have focused on phenotypic divergence using such integrative approaches in the Burmese amber.

Shell-bearing organisms are a model system to investigate morphological evolution because of the high preservation quality of shells as fossils and the ease of morphological quantification^6^. For example, the fossil record of shelled gastropods document diversification with marked changes of shell morphologies over time in some clades^6,7^, while also showing high morphological conservatism in some lineages through the Mesozoic and Paleozoic^8,9^. In gastropods, terrestrial invasion is recognized in at least three major groups: Neritimorpha, Caenogastropoda, and Heterobranchia^10,11^. Based on fossil records and molecular phylogenetic analyses of living species^12,13^, most terrestrial mollusks, also known as land snails, gradually diversified during the middle Mesozoic. The fossil records of most gastropods can be well preserved, but compared with marine mollusks that have thick and hard shells, fossils of land snails are relatively rare due to thin and fragile shells. Gastropods also have other characteristic traits that are useful in delimiting extant species, including the operculum, periostracum, and soft internal organs. The operculum protects soft tissue from dry conditions and predators^14^, while the periostracum is a thin chitonized outer layer on the shell’s surface. Soft parts of the body also show differences in ecologically relevant traits such as digestive systems among groups. However, when and how such traits evolved is poorly understood because of the difficulty of finding fossils with well-preserved soft tissues.

One clade of snails, Caenogastropods, form a notably successful group, possibly accounting for 30–50% of land snail diversity in tropical and subtropical areas^15^. Within Caenogastropoda, the superfamily Cyclophoroidea forms widespread modern terrestrial fauna^16^, comprised of the families Cyclophoridae, Diplommatinidae, and Pupinidae: all common land snails with a chitonized operculum^17^. The earliest known fossils of the extant genera of Cyclophoridae (*Alycaeus* and *Cyclophorus*) and Pupinidae (*Tortulosa*) were recently recorded in Vietnam (the earliest Miocene, Aquitanian 23–21 Ma^16^ as well-preserved shells). However, fossil records of these Cyclophoroidea are rare, especially in the Mesozoic or early Cenozoic (Table S1, there is also some uncertainty as to the identity of some of these early putative cyclophoroideans^16^), and almost all these specimens are limited to shells, so the evolution of other traits remains unclear.

Recent paleontological studies have reported well-preserved fossils of Cyclophoridae, Diplommatinidae, and Pupinidae from Burmese amber^18–20^. In particular, Xing *et al*.^19^ described the fossilized tentacle and an eye of a juvenile cyclophorid from Burmese amber, and identified a potentially preserved operculum. These studies indicate the presence of a mid-Cretaceous land snail and the potential for the preservation of soft body structure when fossilized in amber. However, detailed information regarding other structures (e.g., the operculum and periostracum) is lacking and studies of how these structures might have evolved over time are limited^18–20^.

In the present study, nine fossils of mid-Cretaceous operculate land snails in excellent condition were collected from Burmese amber, including the diplommatinid genus *Euthema* described in a previous study^18^. The shell morphologies of some of these fossils are similar to that of the extant genera of the Cyclophoroidea. The fossils we described here are characterized not only by a range of a well-preserved shell, but also in some cases by a operculum and periostracum, soft anatomical characters, and even excrements. Through micro-CT scanning, we present the first instance of the morphology of a chitonized operculum from the Mesozoic. While phylogenetic studies have been carried out of a few modern groups^15,21–23^, the timing of divergences among major cyclophoroidean groups remains unclear. So we estimate the divergence time of the Cyclophoroidea for the first time using both fossil calibrations and molecular data. Finally, we discuss the evolutionary history of the Cyclophoroidea, and show how the newly discovered fossils are morphologically similar to modern taxa and how they offer crucial information to understand phenotypic evolution within the Cyclophoroidea.

## Results

### Divergence time estimation

**Figure 1:**
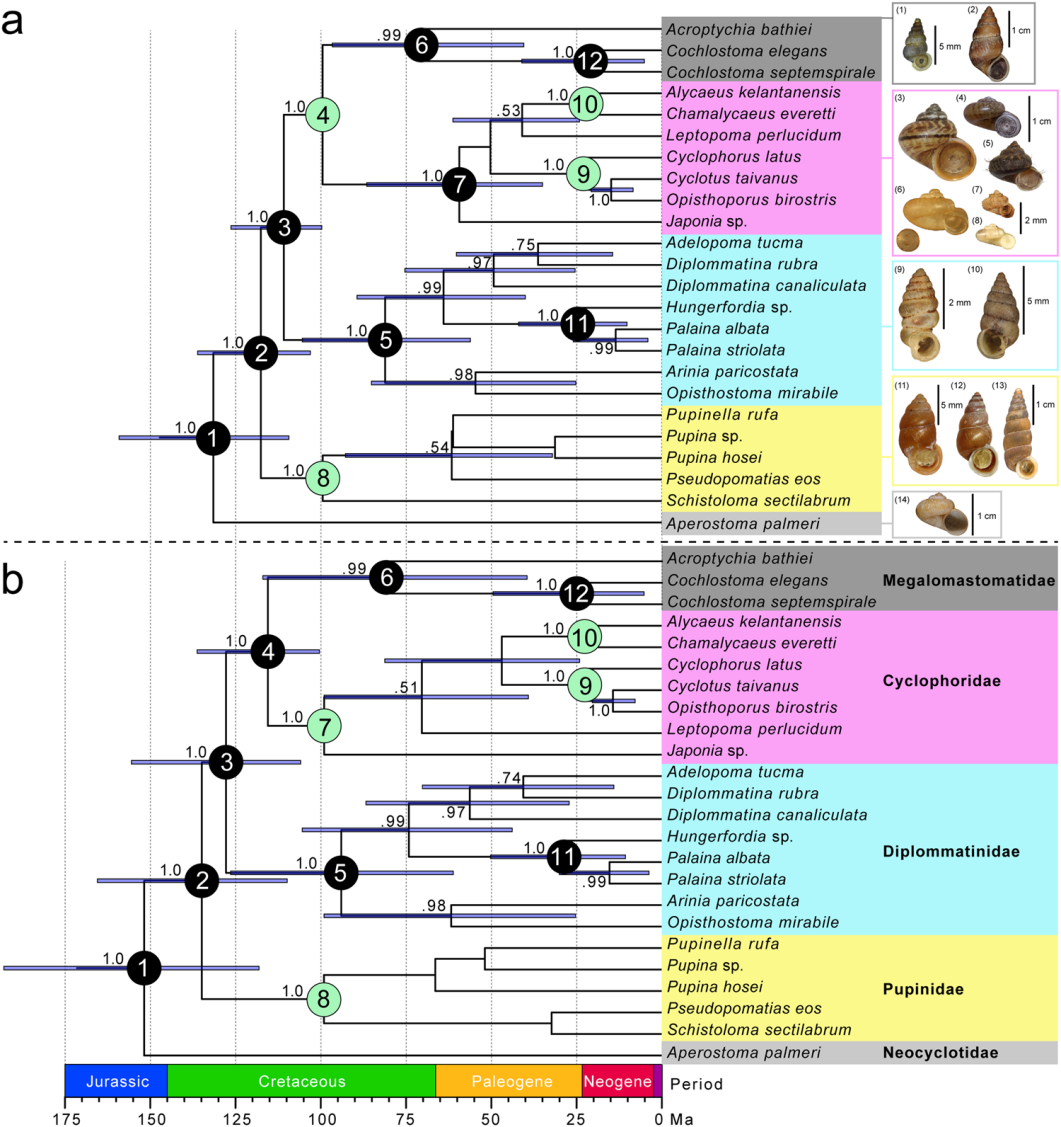
Maximum clade credibility trees generated with the BEAST2 analysis the combined sequences (COI, 16 S, and H3 genes), and the shell morphologies and excrements of the extant representative of the Cyclophoroidea. The outgroups (*Conus* and *Pomacea*) are not shown. For convenience, we assign numbers to the major nodes (Table S2). Numbers on branches indicate Bayesian posterior probabilities. The node bars indicate a 95% CI for the divergence times. The principal nodes are named by nominal numbers. The green circles indicate the calibration points. (**a**) method A and (**b**) method B were calculated using fossil calibration. The photographs of shells in (**a**) represent each family [Megalomastomatidae: (1) *Cochlostoma achaicum coeruleum*, (2) *Hainesia litturatum*; Cyclophoridae: (3) *Cyclophorus oshimanus*, (4) *Cyclotus campanulatus*, (5) *Lagocheilus* sp., (6) *Chamalycaeus laevis*, (7) *Cyathopoma africanum*, (8) *Nakadaella micron*; Diplommatinidae: (9) *Diplommatina immersidens*, (10) *Diplommatina electa*; Pupinidae: (11) *Pupinella rufa*, (12) *Schistoloma sectilabrum*, (13) *Pseudopomatias maasseni*; Neocyclotidae: (14) *Aperostoma burringtoni*]. The scale bars indicate each shell size [(3) to (5): 1 cm, (6) to (8): 2 mm, (12) and (13): 1 cm]. For convenience, the family Alycaeidae is treated as Cyclophoridae based on Webster *et al*.^22^.

We used two estimates for the basal divergence within Cyclophoroidea: fossil species of Cyclophoridae from Burmese amber were treated as a calibration point for the minimum constraint of node 4, and Pupinidae from Burmese amber were used for a calibration point for the minimum constraints of node 8 (Method A: Fig. 1a); each Cyclophoridae and Pupinidae from Burmese amber were used for two calibration points for the minimum constraints of nodes 7 and 8 (Method B: Fig. 1b). In addition, divergence times were calculated using molecular clock rate of COI (Method C: Fig. S1), using three different analyses (Methods A–C, see the Material & Methods; Figs 1 and S1). The ESS values produced in Tracer v. 1.6 exceeded 200 for all parameters. The inferred Bayesian phylogenetic relationships obtained from the analyses performed in BEAST2 are shown in Figs 1a,b and S1.

In Method A (Fig. 1a), the mean time of the first divergence of the modern families of the Cyclophoroidea (node 1) was estimated to be 131 Ma (95% HPD interval 109–159 Ma). In addition, the mean times of divergence of Diplommatinidae (node 5), Megalomastomatidae (node 6), and Cyclophoridae (node 7) were estimated to be 81 Ma, 70 Ma, and 59 Ma, respectively. It appears that the origin of nearly all families occurred in the Cretaceous and Paleogene.

In Method B (Fig. 1b), the mean time of the first divergence of the modern families of the Cyclophoroidea (node 1) was estimated to be 152 Ma (95% HPD interval 118–193 Ma). In addition, the mean times of divergence of Diplommatinidae (node 5) and Megalomastomatidae (node 6) were estimated to be 94 Ma, and 81 Ma, respectively. According to this method, the mean time of the first divergence of the modern families of the Cyclophoroidea is in the Jurassic to Cretaceous, but it appears that the origin of nearly all families occurred in the Cretaceous and Paleogene.

In contrast, using the molecular clock rate of COI (Method C: Fig. S1), the mean time of the first divergence of the modern families of the Cyclophoroidea (node 1) was estimated to be much younger, namely 91 Ma (95% HPD interval 69–116 Ma). The mean times of divergence of Diplommatinidae (node 5), Megalomastomatidae (node 6), Cyclophoridae (node 7), and Pupinidae (node 8) were estimated to be 62 Ma, 55 Ma, 60 Ma, and 51 Ma, respectively. In addition, the mean times of divergence of nodes 4, 9, and 10 were 73 Ma, 48 Ma, and 40 Ma. Thus, the modern families mostly diversified in the Cretaceous and Paleogene. Additional information of the results of the divergence time estimation is shown in Table S2.

### Systematic paleontology

Superfamily: Cyclophoroidea Gray, 1847

Family: Cyclophoridae Gray, 1847

Genus: *Archaeocyclotus* Asato and Hirano, gen. nov.

#### LSID (Life Science Identifier)

urn:lsid:zoobank.org:act:BD3CBBF6-BECD-4BBF-91E6-832530C63FBD.

#### Type species

*Archaeocyclotus plicatula* Asato and Hirano, sp. nov, here designated.

#### Diagnosis

Minute, thin and discoidal shell characterized by a phaneromphalous shell base and an inflated spire whorl with deeply impressed suture. Shell surface ornamented with fine but prominent plica-like ribs and growth lines, partly covered by very short periostracal hairs.

#### Etymology

The generic name is a combination of *Archaeo*- (meaning ancient in classical Greek) and *Cyclotus*, the extant cyclophorid genus most similar to the new taxon.

#### Remarks

Mesozoic to early Cenozoic fossils of Cyclophoridae are only known from six species in one genus, *Palaeocyclophorus* Wenz, 1923 (see below remarks of *A*. *plicatula*). This extinct genus can be clearly distinguished from *Archaeocyclotus* Asato and Hirano, gen. nov. by having a middle, thick and trochiform shell with an angulation at the middle portion of whorl.

According to the records of modern land snails, 22 genera of the cyclophorids are distributed throughout Southeast Asia^24–28^. *Pterocyclos* Benson, 1832, *Platyrhaphe* Möllendorff, 1890, *Cyclotus* Swainson, 1840, *Aulopoma* Troschel, 1847, *Scabrina* Blanford, 1863, *Theobaldius* Nevill, 1878, *Lagocheilus* Blanford, 1864 (low-spired species), *Micraulax* Theobald, 1876, *Crossopoma* Pfeiffer, 1847 and *Ptychopoma* Möllendorff, 1885 are similar in shell morphology to *Archaeocyclotus* Asato and Hirano, gen. nov. in possessing a very depressed turbinate shell and appreciably wide umbilicus without any breathing sutural tube. However, in each of these extant genera, the shell is much larger with a smooth surface, ornamented by growth lines and obscure spiral costa/cords. The summary of morphological comparisons between these extant genera and *Archaeocyclotus* Asato and Hirano, gen. nov. is compiled in Table 1.

**Table 1:** Morphological comparison between *Archaeocyclotus* Asato and Hirano, gen. nov. and some morphologically similar genera of Cyclophoridae.

Genus	Morphological characters						
	Size (mm)	Texture	Shell form	Base	Umbilicus	Aperture	Ornamentations
*Cyclotus* Swainson, 1840	Small to middle (10–30)	Thin, glossy	Discoidal to very low-turbiniform	Phaneromphalous	Wide, shallow	Circular	Smooth, growth lines
*Pterocyclos* Benson, 1832	Small to middle (10–31)	Thin, glossy	Discoidal to very low-turbiniform	Phaneromphalous	Wide, shallow	Circular	Smooth, growth lines
*Platyrhaphe* Möllendorff, 1890	Small (5–10)	Thin, glossy	Discoidal to very low-turbiniform	Phaneromphalous	Wide, shallow	Circular	Smooth, growth lines
*Aulopoma* Troschel, 1847	Small to middle (10–30)	Thin, glossy	low-turbiniform	Phaneromphalous	Narrow, deep	Circular	Smooth, growth lines
*Scabrina* Blanford, 1863	Small to middle (10–31)	Thin, glossy	Discoidal to very low-turbiniform	Phaneromphalous	Wide, shallow	Circular	Smooth, growth lines with periostracal hairs
*Theobaldius* Nevill, 1878	Small to middle (10–31)	Thin, glossy	Discoidal to very low-turbiniform	Phaneromphalous	Wide, shallow	Circular	Smooth, growth lines
*Lagocheilus* Blanford, 1864	Small (5–10)	Thin, solid	Low-trochiform to trochiform	Phaneromphalous	Narrow, deep	Circular	Smooth, growth lines with periostracal hairs
*Micraulax* Theobald, 1876	Small to middle (10–31)	Thin	low-turbiniform	Phaneromphalous	Narrow, deep	Circular	Smooth, growth lines
*Crassopoma* Pfeiffer, 1847	Small to middle (10–32)	Thin, glossy	Discoidal to very low-turbiniform	Phaneromphalous	Wide, shallow	Circular	Smooth, growth lines
*Ptychopoma* Möllendorff, 1885	Small to middle (10–33)	Thin	Discoidal to very low-turbiniform	Phaneromphalous	Wide, shallow	Circular	Smooth, growth lines
*Archaeocyclotus* Asato and Hirano, gen. nov.	Minute (around 5)	Thin, solid	Discoidal	Phaneromphalous	Wide, shallow	Ovoid to elliptical	Fine growth ribs with short periostracal hairs

*Archaeocyclotus plicatula* Asato and Hirano, sp. nov.

Figure 2

**Figure 2:**
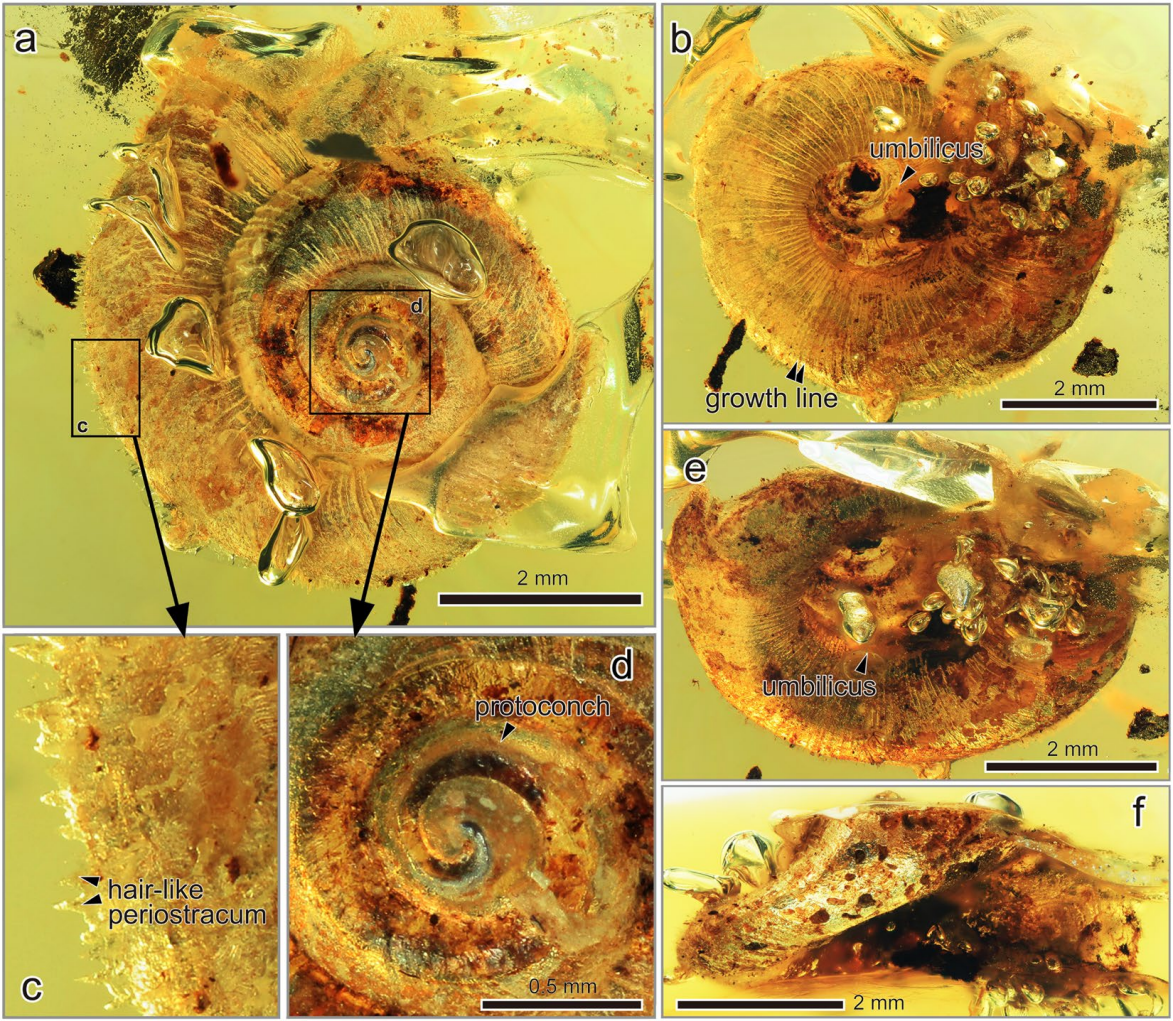
*Archaeocyclotus plicatula* Asato and Hirano, gen. et sp. nov. from the mid-Cretaceous Burmese amber. (**a**–**f)** Holotype NMNS PM 27992. (**a)** Apical view showing a protoconch, growth ribs and periostracum. (**b)** Ventral view showing a large umbilicus, growth ribs and lines. (**c)** Close-up of square (**c)** in (**a)**, showing hair-like periostracum. (**d)** Close-up of the square (**d)** in (**a)**, showing a protoconch. (**e)** Oblique ventral view showing a large and deep umbilicus and growth ribs. (**f)** Apertural view showing a shell form.

*LSID (Life Science Identifier)*: urn:lsid:zoobank.org:act:AAA15DC3-C23B-46F9-952D-EA02796C0B9B.

#### Diagnosis

As for the genus.

#### Description

Shell minute and thin, solid, discoidal and slightly keeled, holotype approximately 2.3 mm high and 5.2 mm wide (NMNS PM 27992). Spire about half of length of shell height with a spire angle of 140°–150°. Spire whorls curved and inflated with a deep and impressed suture. First and second whorls wrinkled, last whorl wider, about two thirds of shell height with an almost vertical outer face of whorl. Base not flattened and phaneromphalous (Fig. 2b,e). Aperture ovoid or elliptical. Peristome not continuous. Outer lip rounded, and inner lip slightly reflected near umbilicus. Umbilicus completely open, wide and shallow, about one third of diameter of shell. Periostracum intact, horny and shiny in appearance with hair-like protrusion on collabral lirae (Fig. 2a,c). Surface with very fine axial ribs after whorls; these ribs resemble growth lines (Fig. 2a,b). Growth line prosocline about 50° angle from whorl outline.

#### Locality and horizon

Burmese amber from the Hukawng Valley (26°15′N, 96°34′E), Kachin State, northern Myanmar; lowermost Cenomanian (ca. 99 Ma)^29^, Upper Cretaceous.

#### Material

Holotype, Data label: NMNS PM 27992. Deposited in National Museum of Nature and Science, Tsukuba, Ibaraki, Japan (NMNS).

#### Etymology

The specific name *plicatula* comes from the ornamentation of this species with very fine axial ribs like “plica” on the whorls.

#### Remarks

Mesozoic fossil records of cyclophorid or related taxa are only six species (all in the genus *Palaeocyclophorus*) have been described to our knowledge. *Palaeocyclophorus helicinaeformis* (Boissy, 1848) has a strong keel on the middle of the whorl, but is larger and rounder than *Archaeocyclotus plicatula* gen. et sp. nov.). *Palaeocyclophorus heliciformis* (Matheron, 1832), *P*. *heberti* Roule, 1884, *P*. *luneli* (Matheron, 1842), *P*. *galloprovincialis* (Matheron, 1842), and *P*. *solarium* (Matheron, 1842) were described from the Maastrichtian deposits of France (Table S1) and all are considerably larger than *A. plicatula* gen. et sp. nov. with different shell ornamentations: *P*. *luneli*, *P*. *galloprovincialis*, and *P*. *heliciformis* have a smooth shell surface and *P*. *heberti* and *P*. *solarium* have spiral ribs. These features are different from those observed in *A. plicatula* gen. et sp. nov.

Genus: *Lagocheilus* Blanford, 1864

#### Type species

*Cyclostoma scissimargo* Gould, 1859.

#### Remarks

Our specimens, NSMT PM 28272 and 28273, have small, trochiform and globose shells covered with several periostracal hairs and a circular aperture in which the outer lip is not reflected. These characters are consistent the diagnosis of *Japonia* Gould, 1859 and *Lagocheilus* Blanford, 1864. According to several systematic revision of the land snails^30–33^, *Lagocheilus* Blanford, 1864 has been widely used for Southeast Asian species of *Japonia* Gould, 1859 because *Japonia* was established without any morphological examinations and the type materials of *Japonia* have been lost, so some authors consider *Japonia* to be nomen dubious^34–36^. Although the status of *Japonia* has not been resolved, it may be preferable to use *Lagocheilus*.

The fossil record of *Lagocheilus*/*Japonia* contains only one species, *Japonia* (*Lagocheilus*) *trilirata* (Pfeiffer, 1852), from the late Miocene of Indonesia^37^. Therefore, our specimen is the oldest fossil record of *Lagocheilus*/*Japonia*. This shows that *Lagocheilus* has already appeared in the middle Cretaceous.

*Lagocheilus cretaspira* Asato and Hirano, sp. nov.

Figures 3a–d, S2a

**Figure 3:**
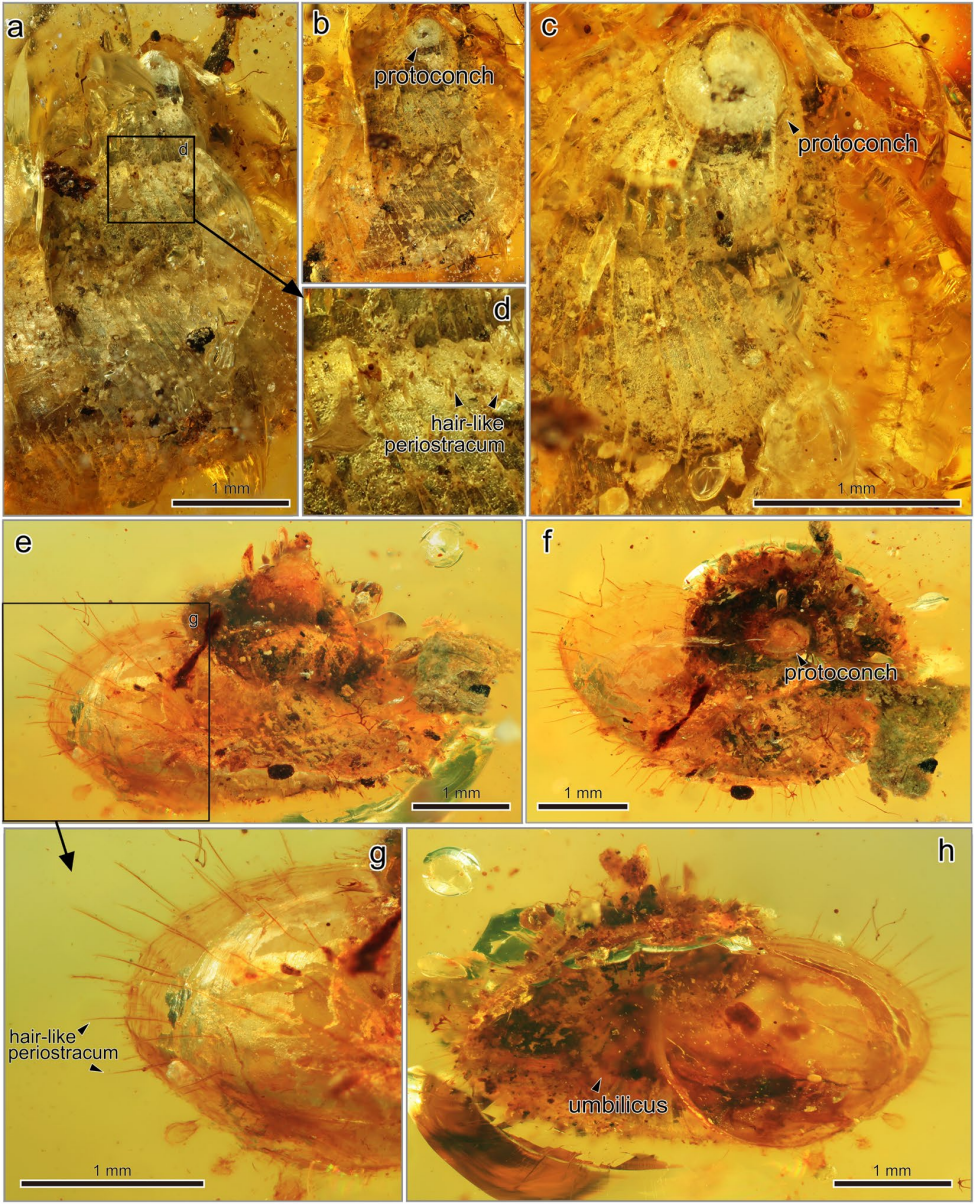
*Lagocheilus cretaspira* Asato and Hirano, sp. nov. and *L*. *electrospira* Asato and Hirano, sp. nov. from the mid-Cretaceous Burmese amber. (**a**–**d**) Holotype of *L*. *cretaspira* (NMNS PM 28272). (**a)** Abapertural view showing hair-like periostracum. (**b)** Apical view showing a protoconch. (**c)** Close-up of apical showing a protoconch. (**d)** Close-up of square (**d)** in (**a)** showing hair-like periostracum. (**e**–**h)** Holotype of *L*. *electrospira* (NMNS PM 28273). (**e)** Abapertural view showing long hair-like periostracum. (**f)** Apical view showing a protoconch. (**g)** Close-up of square (**g)** in (**e)** showing long hair-like periostracum. (**h)** Apertiral view showing an umbilicus.

#### LSID (Life Science Identifier)

urn:lsid:zoobank.org:act:FECC1636-D383-488C-8060-5AB6C81B4D51.

#### Diagnosis

Small, thin, trochiform and globose shell characterized by convex shell whorls with an angulation at lower portion ornamented fine and prominent plica-like collabral lirae furnished with several periostracal hairs (tapering in width toward tips). Umbilicus open and deep. Aperture very nearly circular, outer lip not reflected.

#### Description

Shell small and thin, 4.3 mm in length and 4.2 mm in width. Shell trochiform and conical-globose shape, with moderately convex whorls 4–4.5 in number. An angulation at lower portion of body whorl (Fig. S2). The shell sculpture consists of prominent, widely spaced collabral lirae ornamented with short periostracal hairs; indistinct traces of spiral lirae in a few places (Fig. 3a,c). 10–12 rows of periostracal hairs between suture and penultimate whorl, tapering toward their tips (Fig. 3c,d). Periostracum horny and glossy, largely intact and hairy. Umbilicus open and deep (Fig. S2). Aperture nearly circular. Peristome continuous, and outer lip not reflected (Fig. S2).

#### Locality and horizon

Burmese amber from the Hukawng Valley (26°15′N, 96°34′E), Kachin State, northern Myanmar; lowermost Cenomanian (ca. 99 Ma)^29^, Upper Cretaceous.

#### Material

Holotype, Data label: NSMT PM 28272. Deposited in NMNS.

#### Etymology

The specific name *cretaspira* comes from the Cretaceous age of the fossil and the old Greek *spira* meaning spire.

#### Remarks

According to the Mesozoic fossil records of cyclophorid and related taxa, *Lagocheilus cretaspira* sp. nov. is comparable with two Santonian species from Austria, *Leptopoma* (*Trocholeptopoma*?) *cretaceum* Hrubesch, 1965 and *Leptopoma* (*Trocholeptopoma*?) *minutum* Hrubesch, 1965 in having a prominent and deep umbilicus, a continuous and unreflected outer lip and small shell size. However, in these Santonian species, the whorl profile is straight so that the shell is trochoform rather than turbiniform, which can be clearly differentiated from *L*. *cretaspira* sp. nov.

As shown above, *Japonia* (*Lagocheilus*) *trilirata* (Pfeiffer, 1852) is the only species found as a fossil record of *Lagocheilus* and *Japonia*, which was discovered from the late Miocene of Indonesia^37^. *J*. (*L*.) *trilirata* is clearly differentiated from *L*. *cretaspira* sp. nov. as having spiral costa and a flare-like peristome.

The most similar species to *Lagocheilus cretaspira* sp. nov. is *L. electrospira* sp. nov., another new species of *Lagocheilus* Gould, 1859 from the Cretaceous Burmese amber (see below description), which has a trochiform shell, an unreflected outer lip and a prominent and deep umbilicus. In the latter species, however, the shell surface is smooth and the periostracal hairs are simpler and longer than those of *L*. *cretaspira* sp. nov.

*Lagocheilus electrospira* Asato and Hirano, sp. nov.

Figures 3e–h, S2b

#### LSID (Life Science Identifier)

urn:lsid:zoobank.org:act:0BE60950-1288-4BB1-96F7-C2061A24A44B.

#### Diagnosis

Small, thin, low-trochiform and globose shell characterized by convex shell whorls furnished slender and long periostracal hairs. Shell surface smooth. Umbilicus open and deep. Aperture very near circular, outer lip not reflected.

#### Description

Shell small and thin, 2.6 mm in length and 4.0 mm in width. Shell turbiniform and conical-globose shape, with moderately convex whorls 3.5–4 in number. Body whorl slightly flattened because of compaction (Figs 3h and S3). Shell surface smooth, ornamented only oblique growth lines covered by slender periostracal hairs (Fig. 3e,g). 8–9 long rows of periostracal hairs between suture and penultimate whorl, becoming shorter length near shell base (Fig. 3g,h). Umbilicus open and deep (Figs 3h and S3). Aperture nearly circular. Peristome continuous, and outer lip not reflected (Fig. 3h).

#### Locality and horizon

Burmese amber from Hukawng Valley (26°15′N, 96°34′E), Kachin State, northern Myanmar; lowermost Cenomanian (ca. 99 Ma)^29^, Upper Cretaceous.

#### Material

Holotype, Data label: NSMT PM 28273. Deposited in NMNS.

#### Etymology

The specific name is a combination of *electro*- (meaning an amber in Greek) and *spira*- (meaning spire).

#### Remarks

For comparisons between this species and other Mesozoic cyclophorid species, see the remarks of *Lagocheilus cretaspira* Asato and Hirano, sp. nov.

The most similar species to *Lagocheilus electrospira* sp. nov. is *L*. *cretaspira* sp. nov. Both species have a trochiform shell, an unreflected outer lip and a prominent and deep umbilicus. In the latter species, however, the shell surface is ornamented with prominent growth lirae. Also, the periostracal hairs of *L*. *electrospira* sp. nov. are simpler and longer than those of *L*. *cretaspira* sp. nov. These characters clearly distinguish *L*. *electrospira* sp. nov. from *L*. *cretaspira* sp. nov.

Family: Diplommatinidae Pfeiffer, 1856

Genus: *Euthema* Yu, Wang & Pan, 2018.

#### Type species

*Euthema naggsi* Yu, Wang & Pan, 2018

#### Diagnosis

Very small with nearly-cylindrical shell, dextral, consisting of about 5–6 regularly increasing whorls. Whorl low and wider than high, penultimate whorl coiling tight. Surface ornamented with nearly S-shape and prominent collabral lirae. Aperture nearly round. See details: Yu *et al*.^18^.

#### Remarks

This monotypic genus was described based on a specimen from Burmese amber^18^.

*Euthema hesoana* Asato and Hirano, sp. nov.

Figure 4

**Figure 4:**
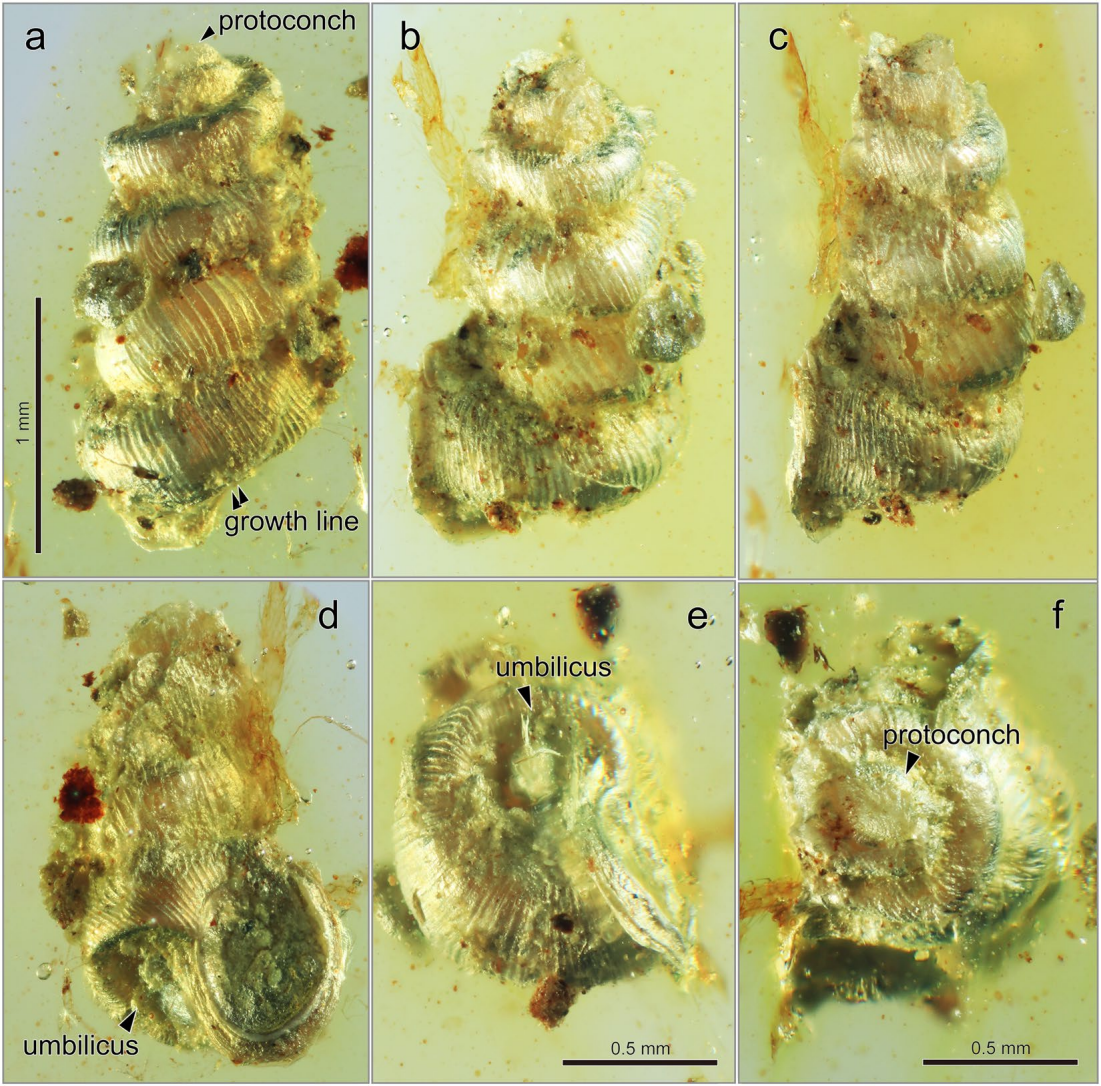
*Euthema hesoana* Asato and Hirano, sp. nov. from the mid-Cretaceous Burmese amber. (**a**–**f)** NMNS PM 28274. (**a)** Abapertural view showing a protoconch and growth lines. (**b)** Oblique abapertural view. (**c)** Oblique abapertural view. (**d)** Apertural view showing umbilicus and an aperture. (**e)** Basal view showing umbilicus. (**f)** Apical view showing a protoconch.

#### LSID (Life Science Identifier)

urn:lsid:zoobank.org:pub:49715B29-C5CB-4A9B-883D-2CB3F6D7661F.

#### Diagnosis

Very small with nearly-cylindrical shell, dextral, consisting of about 5–6 regularly increasing whorls. Whorl low and wider than height, penultimate whorl coiling tight. Surface ornamented with nearly S-shaped and prominent collabral lirae. Aperture nearly round. Shell base prominent periumbilical keel with moderately deep and wide umbilicus.

#### Description

Shell very small and dextral, nearly cylindrical in shape, around 2.0 mm high, and 1.0 mm wide. 5–6 whorls, slightly angulated and inflated (Fig. 4a–d). Penultimate whorl tightly coiled. Apex blunt. Surface ornamented by nearly S-shape and strongly prominent collabral lirae (Fig. 4b,d). Suture deeply impressed with abutting whorls. Aperture sub-circular, and apertural margin broadened and strongly reflected (Fig. 4b,c). Out lip arched-round with rounded-angular in posterior and widely rounded in anterior. Peristome thickened and expanded, and flaring edge slightly curved, i.e., not in one plane (Fig. 4d). Shell base has a very prominent, ribbed periumbilical keel; only growth lines present. Umbilicus moderately deep and wide (Fig. 4d,e).

#### Locality and horizon

Burmese amber from the Hukawng Valley (26°15′N, 96°34′E), Kachin State, northern Myanmar; lowermost Cenomanian (ca. 99 Ma)^29^, Upper Cretaceous.

#### Material

Holotype, Data label: NMNS PM 28274. Deposited in NMNS.

#### Etymology

The specific name is derived from “heso-ana” meaning an umbilicus in Japanese, which is a typical diagnostic character of this species.

#### Remarks

Our specimen NMNS PM 28274 has a small, nearly-cylindrical and dextral shell with tightly coiling penultimate whorl ornamented by S-shaped collabullar ribs. These characters suggest that this species belongs to *Euthema*^18^. *Euthema naggsi* Yu *et al*., 2018 is the only species in this genus, and is clearly differentiated from *Euthema hesoana* Asato and Hirano, sp. nov. by having a round and inflated shell base with a shallow and narrow umbilicus.

Family: Pupinidae Pfeiffer, 1853

Genus: *Schistoloma* Kobelt, 1902.

#### Type species

*Schistoloma alta* (Sowerby, 1842).

#### Remarks

Mesozoic to early Cenozoic fossil records of Pupinidae are classified in five extinct genera (*Ischurostoma* Bourguignat, 1874, *Rognacia* Oppenheim, 1895, *Kallomastoma* Stache, 1889, *Ventriculus* Wenz, 1914 and *Cyclomastoma* Hrubesch, 1965) and one modern genus (*Pseudopomatias*? Möllendorff, 1885) from Europe and Burmese amber (Supplementary Text; Table S1).

Our specimen NSMT PM 27993 has a small, pupiniform shell with a wide, angled parietal-palatal transition of the aperture and a closed umbilicus, whose characters are consistent the diagnosis of *Schistoloma* according to Páll-Gergely *et al*.^38^. Moreover, *Schistoloma electrothauma* sp. nov. has a thin operculum ornamented by densely sinistral cord that is mostly similar to the tendency of the operculum in Pupinidae (e.g., Páll-Gergely *et al*.^15^). To our knowledge, *S*. *electrothauma* sp. nov. is the first fossil species of *Schistoloma*, showing that *Schistoloma* appeared in the middle Cretaceous.

*Schistoloma electrothauma* Asato and Hirano, sp. nov.

Figure 5, Video S1

**Figure 5:**
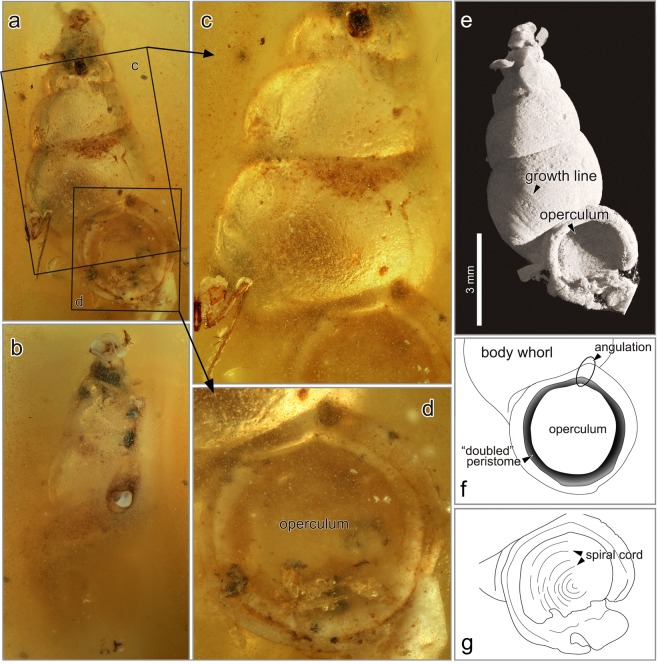
*Schistoloma electrothauma* Asato and Hirano, sp. nov. from the mid-Cretaceous Burmese amber. (**a**–**e)** Holotype NMNS PM 27993. (**a)** Apertural view. (**b)** Abapertural view. (**c)** Close-up square (**c)** in (**a)** showing the shell surface. (**d)** Close-up square (**d)** in (**a)** showing an aperture and an operculum. (**e)** CT scanning picture of the apertural view showing growth lines and an operculum. (**f**,**g)** Sketches of the aperture. (**f)** Line drawing of the aperture showing a doubled peristome and an angulation near junction of inner lip and parietal wall. (**g)** Line drawing showing an operculum with spiral cords. Copyright © 2017 Shimadzu Corporation for **(e)**.

#### LSID (Life Science Identifier)

urn:lsid:zoobank.org:act:7E3B11FE-0E83-45E8-85BC-5E5A71BE483E.

#### Diagnosis

Shell small, turriform/pupiniform shell without any ribs, ornamented by only growth lines becoming a robust and rough shell surface. Aperture circular with a thickened peristome, and doubled with a short and obscure angulation near junction of inner lip and parietal wall. Operculum having a thin lamella ornamented by a multispiral and sinistral cord.

#### Description

Shell small, turriform to slightly pupiniform, around 9.6 mm high, and 4.5 mm wide in holotype (Fig. 5a,e). 5–6 whorls, slightly curved and inflated. Transverse growth lines robust as growth ribs near aperture (Supplementary video S1). Suture slightly impressed with abutting whorls. Apex acute and regularly torted, and a spire angle 47° in holotype. Aperture circular with a right posterior triangular/drop-like shape, a short and obscure angulation near junction of inner lip and parietal wall (Fig. 5d,f). Peristome thickened and expanded, and flaring edge slightly curved, i.e., not in one plane (doubled peristome in Fig. 5f). Shell base well rounded and smooth; growth lines present (Fig. 5c,e). Umbilicus narrow and linear. Operculum not calcified (probably chitonized), lamellate, thin and multispiral (Fig. 5g).

#### Locality and horizon

Burmese amber from Hukawng Valley (26°15′N, 96°34′E), Kachin State, northern Myanmar; lowermost Cenomanian (ca. 99 Ma)^29^, Upper Cretaceous.

#### Material

Holotype, Data label: NSMT PM 27993. Deposited in NMNS.

#### Etymology

The specific name is a combination of two Greek words: *electro*, meaning amber and *thauma*, which means miracle since the inclusion of this holotype in Cretaceous amber with such extraordinarily well preservation is exceptional.

#### Remarks

According to the records of *Schistoloma*, 16 species are distributed in ten countries (Table S3). Based on the shell features, five of these species were available for comparison with *Schistoloma electrothauma* sp. nov., but all species are much bigger than *S*. *electrothauma*, except for *S*. *inermis* (Bavay & Dautzenberg, 1909). The shell size of *S*. *inermis* is approximately 15 mm in length by 7 mm in width^38^, whereas those of *S*. *electrothauma* sp. nov. are only about a half of that size. The two Bornean species, *S*. *anostoma* (Benson, 1852) and *S*. *doriae* (Issel, 1874), are similar to *S*. *electrothauma* sp. nov. in terms of a turriform/pipuniform shell with a thick and doubled peristome. However, in these two species the last whorl is bigger and more rounded than *S*. *electrothauma* sp. nov., which makes the shell wider.

*Schistoloma altum* (Sowerby, 1842) is also similar to *Schistoloma electrothauma* sp. nov. It has a turriform/pupiniform shell profile with a doubled peristome and a narrow umbilicus; however, *S*. *alta* has a “posterior groove” near the junction of the inner lip and the parietal wall, and a well-developed peristome. The Sumatran species *S*. *sumatranum* (Dohrn, 1881) resembles to *S*. *electrothauma* sp. nov. in terms of shell profile and smooth shell ornamentations, but is much wider with more inflated whorls than *S*. *electrothauma* sp. nov. and has a much more developed peristome.

*Schistoloma sectilabrum* (A. Gould, 1844) is probably the species closest to *Schistoloma electrothauma* sp. nov. based on its smooth and turriform/pipuniform shell with a rounded shell base, narrow umbilicus, and doubled peristome. *S*. *sectilabrum* has been recorded in Myanmar, Thailand, and peninsular Malaysia. In *S*. *sectilabrum*, however, the whorl is more inflated and the shell is more turriculate than *S*. *electrothauma* sp. nov.

## Discussion

### Divergence time and evolutionary history

The divergence time estimates using fossil calibration from this study suggest the first divergence within the Cyclophoroidea occurred in the mid-to-late Mesozoic (Jurassic to Cretaceous, Fig. 1). The oldest fossils of the Cyclophoroidea were recorded in the Upper Jurassic to the Lower Cretaceous with brackish and freshwater gastropods (*Loriolina* and *Maillardinus*^39^, hereafter referred to as the “Cyclophoroidea” for convenience). Therefore, since Cyclophoroidea are only known to be terrestrial, it is possible that the oldest “Cyclophoroidea” fossils were erroneously identified. Different dating methods used herein provide mixed support for the ancestry of these fossils. One of our calibration strategies (Method A) suggests that the modern Cyclophoroidea might be a crown group rooted in a single lineage of fossil Cyclophoroidea with all other lineages extinct (Fig. 1a, calibrating nodes 4 and 8). The other (Method B) suggests that these “Cyclophoroidea” fossils might be direct ancestors of modern Cyclophoroidea (Fig. 1b, calibrating nodes 7 and 8).

Almost all families of the extant Cyclophoroidea diversified in the Late Cretaceous and Paleogene. In Method B, the 95% HPD interval of the divergence for Diplommatinidae overlaps 99 Ma (node 5; Fig. 1b). In the case of genus level divergence, our new time estimates based on the fossil calibration overlap with the divergence time of the extant *Aperostoma*^12^. The molecular clock analysis (Fig. S1) suggests divergence times consistent with the oldest fossil records of modern genera of the Cyclophoroidea in Asia (e.g. *Alycaeus* and *Cyclophorus*: ca. 23–21 Ma)^16^. According to prior studies, the COI gene is saturated after about 10 million years, and these same studies have shown evidence of rate variation among species^40^. Therefore, using only the molecular clock rate is potentially underestimating divergence time.

Considering the geographic distribution of the modern Cyclophoroidea, geographical isolation might explain diversification of these snails. For example, the divergence among endemic diplommatinid species of the Belau Islands (node 11) coincides with the oldest estimates for the age of the archipelago (30 Ma)^21^^,41^. In addition, diversification of the extant Cyclophoroidea might stem from other factors. Phylogeny A (Fig. 1a) suggests that family-level diversification occurred near the end of the Cretaceous, aligning with global mass extinctions at that time (Cretaceous-Paleogene boundary^42^, nodes 3–5; Fig. 1a). This rapid diversification coinciding with the K-Pg boundary could stem from reduced ecological constraints if predators and competitors of the ancestral Cyclophoroidea went extinct. Ecological release occurring in areas with unoccupied niches and no predators (often the case in oceanic islands) is hypothesized to have promoted diversification of land snails^43^ and might also explain diversification of the Cyclophoroidea.

### Morphological pattern and evolution of shell

In general, morphology can be affected by physical constraints, (i.e. larger individuals tend to be more affected by gravity than smaller individuals)^44^. The shells of land snails have undergone rapid diversification, reflecting ecological and evolutionary factors such as shifts in habitat^43^, predation^45^ and sexual selection^46^. In addition, different evolutionary patterns of shell morphology at the genus level (long-term stasis and short-term divergence) have been reported^47^. Some extant Cyclophoroidea show remarkably high diversity in shell ornamentation and unique chiral forms^46^ among families. However, the shell morphologies of Cyclophoridae, Diplommatinidae, and Pupinidae fossils are similar to extant species groups, suggesting that the Cyclophoroidea has been under extreme morphological conservatism for nearly 100 million years. Stabilizing selection or developmental constraints^48^ could explain morphological conservatism of shell shape, but we did not attempt to address this in the present study.

A study on fossilized beetles in Burmese amber suggested similar morphological conservatism since the mid-Cretaceous^47^. For example, the *Nicrophorus* beetles found in Burmese amber are 1/4 the length of extant species^49^, although there is a bias of size distribution due to limited body size preserved in amber. The insects inhabit leaf litter in tropical forests, a hidden and stable environment that might have promoted morphological conservatism^49^. The typical habitat of extant Cyclophoroidea is under fallen leaves, logs, and rocks in the forest of tropical regions^21^, so cyclophoroideans may have been inhabiting similar habitats to these beetles since the Cretaceous, which in turn might have favored morphological similarities.

Well-preserved specimens of fossil cyclophorid species (*Alycaeus*, *Cyclophorus*, and *Tortulosa*: 23–21 Ma)^16^ and cyclophoroidean fossils from Burmese amber have similar shell shape to extant species (e.g. there are some extant species of the pupinid genera *Tortulosa* and *Pseudopomatias* that are similar in size to *S*. *electrothauma* sp. nov.^20,50^). However, cyclophoroidean fossils from Burmese amber except for *S*. *electrothauma* sp. nov. are smaller than most extant snails and the above fossil *Alycaeus* and *Cyclophorus* species (Fig. 1, see Systematic Paleontology section)^16^. Cyclophoroidea fossils have similar shell shapes to the extant snails, but still display high diversity in shell size through evolutionary history. Shell shape and size are different features and are likely under different selective pressures. Size might be driven by ecological shifts or sexual selection independently (e.g. Goodfriend^51^, Kimura *et al*.^52^), but the reason for size variation between fossil and extant species warrants further study.

### Morphological pattern and evolution of soft tissue

**Figure 6:**
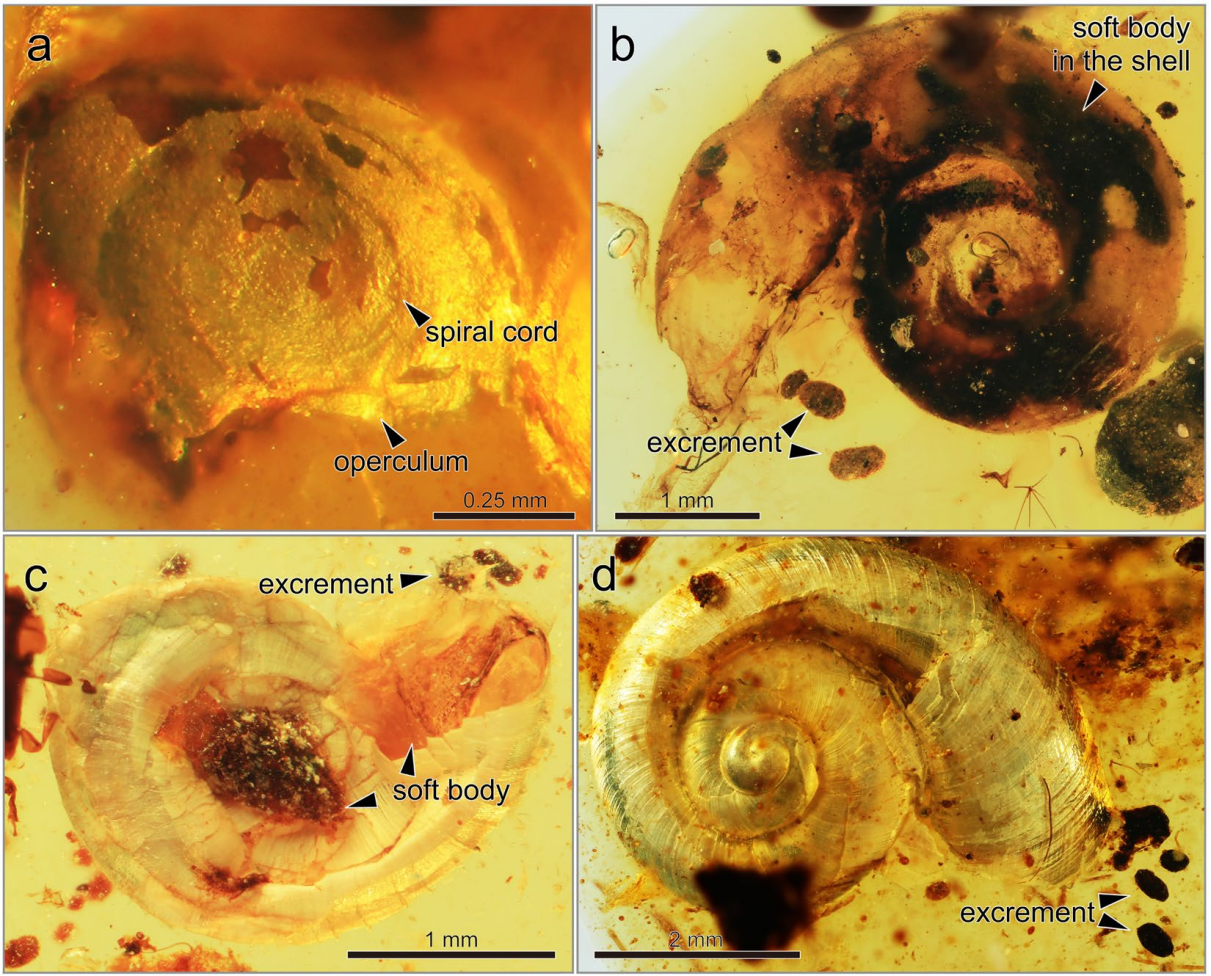
Fossils of operculum, excrements, and soft bodies. **(a)** Aperture close-up of Cyclophoridae sp. 4 (NMNS PM 28278) showing an operculum with a spiral cord. **(b)** Ventral view of Cyclophoridae sp. 1 (NMNS PM 28275) showing a soft body in the shell and elliptical excrements. **(c)** Ventral view of Cyclophoridae sp. 2 (NMNS PM 28276) showing elliptical excrements and soft parts. **(d)** Apical view of Cyclophoridae sp. 3 (NMNS PM 28277) showing growth lines and elliptical excrements.

**Figure 7:**
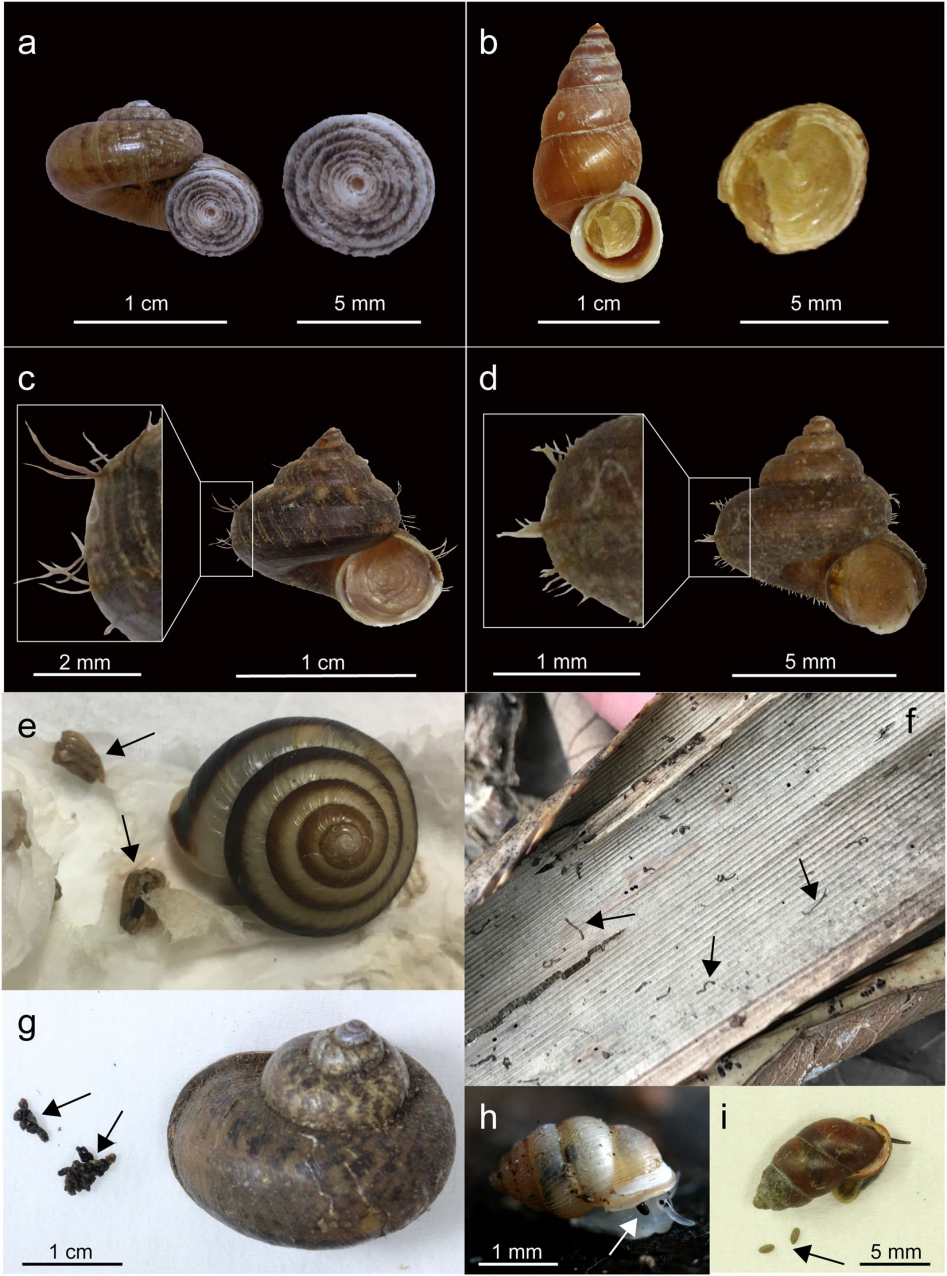
The morphologies of the extant representative of the Cyclophoroidea and comparing excrements among terrestrial mollusks groups. **(a)** Cyclotus campanulatus and its calcareous operculum. **(b)** *Schistoloma sectilabrum* and its chitinous operculum. **(c)** *Lagocheilus* sp. and its long hair-like periostracum. **(d)** *Japonia* sp. and its short hair-like periostracum. **(e)** Rope-like excrements of Heterobranchia: *Euhadra grata*. **(f)** Rope-like excrements of Neritimorpha: *Ogasawarana* sp. **(g–i)** Granular-like excrements of Caenogastropoda (Cyclophoroidea): **(g)** *Cyclophorus oshimanus*, **(h)** *Diplommatina tanegashimae kyushuensis*, **(i)** *Pupinella rufa*. The arrows in **(g–i)** indicate excrements of each individual.

Xing *et al*.^19^ discussed preservation of soft tissues of snail fossils in Miocene Dominican, Eocene Baltic, and Burmese ambers. A prior study documented possible operculum of terrestrial Neritimorpha and an indeterminate family (treated as Prosobranch land snail) in the Miocene Dominican amber, but did not mention if operculum was chitinous or not^53^. Fossilized chitinous operculum and periostracum on the shell surface are very rare, and our fossils are the oldest and most definitive examples of chitinous structures in mollusks (Figs 2, 3, 5, and 6). Fossils of calcareous opercula (possibly of the “Cyclophoroidea”) were recorded in the Upper Jurassic to the Lower Cretaceous^39^. If these fossilized opercula are from true Cyclophoroidea, then the evolutionary pattern of the operculum of the Cyclophoroidea probably started from a calcareous structure. Some extant species of the Cyclophoroidea have a calcareous operculum (Fig. 7a)^17^, and morphological shifts of operculum between calcareous and chitinous may have occurred several times.

Many extant cyclophoroid species have a thin, flat, membranous, and closely coiled operculum^15,17^ (Fig. 1). For example, some pupinid snails have a chitinous operculum (e.g. Fig. 7b) similar to the fossil species (Fig. 5) presented herein that also have a simple chitinous and closely coiled operculum and chitinous periostracum, suggesting ecological characteristics as the extant species. Notwithstanding the material used, morphological change in the operculum in the Cyclophoroidea seems to be limited, at least based on the external morphology observed in the fossil and extant species of this group. Phylogenetic constraints on the operculum may be strong, as the operculum appears to show long-term morphological conservatism.

In land snails, horny and hair-like periostracum has evolved several times among closely related species^54^. Adaptive significance of hair-like periostracum in the Cyclophoroidea is still unclear, but we found morphological similarity of chitinous periostracum between the extant (in particular some of the recent Cyclophoridae) and the Late Cretaceous fossil species (Figs 1–3). Given the similarity in (presumably non-random) patterns of hair-like periostracum in our fossilized individuals and what is observed in extant snails, we are confident that we have identified these structures correctly (Figs 1–3, and 7). Moreover, the variation in hair length of the periostracum we find in our fossilized individuals (long: Fig. 3g, or short: Fig. 3d) is comparable to the variation found in extant species (long: Fig. 7c, or short: Fig. 7d).

Finally, excrements (Fig. 6b–d) and soft bodies (Fig. 6b,c) were preserved along with the shell, chitinous operculum (Figs 5 and 6a), and periostracum (Figs 1–3). Typically, excrements of terrestrial species of Heterobranchia (Fig. 7e) and Neritimorpha (Fig. 7f) are rope-like shapes. According to a prior study, Burmese amber contained excrements found near the aperture of an unidentified snail fossil, indicating that the snail was captured by resin while it was alive^55^. These excrements of both the present and prior studies^55^ are very similar to that of the extant Cyclophoroidea (Fig. 7g–i) in terms of granular feces. Excrements in amber can be good trace fossils for determining the existence of snails, even if the direct morphological traits of snails such as the shell are not preserved. These snail excrements could potentially be used to identify the unidentified species in a prior study^55^, which may be the Cyclophoroidea. Morphological similarity of the excrements between the extant and fossil snail species suggests a similar digestive system.

## Conclusion

We present a new genus and five new species of well-preserved fossil land snails from the mid-Cretaceous Burmese amber, including fossilized soft tissues. The first estimation of divergence time among cyclophoroidean families using molecular and fossil data suggests extreme morphological conservatism of the Cyclophoroidea for about 100 million years. Although the excellent preservation of fossils from Burmese amber seen here helps to illuminate species diversity in land snails^18,19^, more specimens will be needed to further understand the evolutionary history of the Cyclophoroidea and other groups of land snails.

## Materials and Methods

### Fossil samples

Burmese amber (burmite) is one of the most attractive source of data for studying the terrestrial biosphere in the middle Cretaceous. It includes diverse biota from the Cretaceous tropical forest^55^. Our specimens were derived from the Hukawng Valley (26°15′N, 96°34′E), which consists of folded Cretaceous and Cenozoic deposits located in the Kachin State of northern Myanmar^29,55^. Recent U–Pb dating of zircons from the amber matrix indicated the earliest Cenomanian age of Burmese amber (98 ± 0.62 Ma)^29^.

Nine Burmese amber specimens were prepared for observations and are deposited in the Invertebrate Paleontology collection in the Department of Geology and Paleontology, NMNS. The holotype of *Archaeocyclotus plicatula* Asato and Hirano, gen. et sp. nov. (NMNS PM 27992), *Lagocheilus cretaspira* Asato and Hirano, sp. nov. (NSMT PM 28272), *L*. *electrospira* Asato and Hirano, sp. nov. (NSMT PM 28273), *Euthema hesoana* Asato and Hirano, sp. nov. (NSMT PM 28274) and *Schistoloma electrothauma* Asato and Hirano, sp. nov. (NSMT PM 27993) are embedded in a rectangular shape of amber. Similarly, additional specimens (Fig. S3, NSMT PM 28275; Fig. S4, NSMT PM 28276; Fig. S5, NSMT PM 28277; Fig. S6, NSMT PM 28278) are preserved in a small, parallelepiped piece.

### Imaging

All photographs were produced using two different models of the digital cameras (EOS 7D or EOS 80D; Canon Inc., Tokyo) equipped with a macro lens (Canon MP-E 65 mm, F2.8, 1–5x; Canon) with a twin flash light (Canon Macro Twin Lite MT-24EX Flash; Canon). Specimens were completely submerged in clove oil (refractive index: 1.52–1.55) to reduce reflections when they were photographed. A photomontage was created with Combine ZM software (Alan Hadley, Sheffield, U.K.). Line drawings were prepared using Adobe Illustrator CC (Adobe Systems Inc., USA). Photos and drawings were edited and assembled with Photoshop CC (Adobe Systems Inc., USA).

The holotype of *Schistoloma electrothauma* Asato and Hirano, sp. nov. was scanned with a micro-focus X-ray CT system (inspeXio SMX-100CT, Shimadzu Corp., Kyoto) by Mr. Takashi Kushibiki (Shimadzu). Scans produced a three-dimensional output with 6.0 μm voxels under a 160 kV and 70 μA X-ray tube. We used software to model and visualize the data (VGstudio MAX ver. 2.2; Volume Graphics GmbH, Heidelberg). The holotypes of *Lagocheilus cretaspira* Asato and Hirano, sp. nov. and *L*. *electrospira* Asato and Hirano, sp. nov. were scanned using a LaTheta (LCT-100) experimental animal X-ray CT system (ALOKA) owned by the National Museum of Nature and Science, Tokyo. Scans produced a three-dimensional output with 60 μm voxels under a 50 kV and 1 mA X-ray tube. Scanning CT data were modeled and visualized by Dr. Megu Gunji (the Natural Museum of Nature and Science, Tokyo) to use software OsiriX MD ver. 8.5.2 (Pixmeo, Geneva, Switzerland).

### Morphological terminology

Morphological terminology generally followed Cox^56^ and Arnold^57^.

### Nomenclatural acts

This published work and the nomenclatural acts it contains have been registered in ZooBank, the proposed online registration system for the International Code of Zoological Nomenclature (ICZN). The ZooBank LSIDs (Life Science Identifiers) can be resolved and the associated information viewed through any standard web browser by appending the LSID to the prefix ‘http://zoobank.org/’. The LSIDs for this publication are: urn:lsid:zoobank.org:pub:49715B29-C5CB-4A9B-883D-2CB3F6D7661F, urn:lsid:zoobank.org:act:BD3CBBF6-BECD-4BBF-91E6-832530C63FBD, urn:lsid:zoobank.org:act:AAA15DC3-C23B-46F9-952D-EA02796C0B9B, urn:lsid:zoobank.org:act:FECC1636-D383-488C-8060-5AB6C81B4D51, urn:lsid:zoobank.org:act:0BE60950-1288-4BB1-96F7-C2061A24A44B, urn:lsid:zoobank.org:pub:49715B29-C5CB-4A9B-883D-2CB3F6D7661F, and urn:lsid:zoobank.org:act:7E3B11FE-0E83-45E8-85BC-5E5A71BE483E.

### Divergence time estimation

To estimate the divergence time among the families of the Cyclophoroidea, we first obtained GenBank data (mitochondrial cytochrome oxidase subunit 1 (COI) gene, the 16S rRNA region, and Histone 3 (H3)) of 23 species of the Cyclophoroidea and outgroups (*Pomacea insularum* and *Conus miles*^22^; Table S4). Specifics of DNA extraction, PCR, alignment parameters, and model selection are included in Table S5 and Supplementary Text.

Approximate divergence times were estimated using the uncorrelated lognormal relaxed clock model as implemented in BEAST 2.4.5^58^. Divergence times were calculated using two patterns of parameter sets: (1) fossil records (Fig. 1) and (2) molecular clock rate (Fig. S1). To investigate divergence times, we used the phylogenetic relationships from Webster *et al*.^22^ to determine tree topology. This tree topology indicated that Pupinidae diversified earlier than Cyclophoridae^22^. According to the prior studies^18,19^ and the present study, Cyclophoridae and Pupinidae have multiple fossil records in Burmese amber (98.79 ± 0.62 Ma)^29^. Therefore, we treated Cyclophoridae species of these fossils as a direct common ancestor of the modern Cyclophoridae and used these snails as a calibration point for the lower constraint of node 4 (Method A: Fig. 1a). As an alternative method, we treated these multiple fossil snails of Cyclophoridae as a calibration point for the lower constraints of node 7 (Method B: Fig. 1b; the extant Cyclophoridae and Pupinidae had been diversified). According to this and previous studies^20^, the extant Pupinidae had already diversified in the Cretaceous. Therefore, we treated these multiple fossils of Pupinidae as a calibration point for the lower constraint of node 8 in both methods. We also used two fossils of extant genera (*Alycaeus* and *Cyclophorus*) as calibration points for the lower constraint of the divergence time of each genus (23–21 Ma)^16^ in both methods. All fossil calibrations were given an exponential prior distribution (mean = 1, offset = 98.17 for nodes 4 and 8 in Fig. 1a, and nodes 7 and 8 in Fig. 1b; mean = 1, offset = 21 for nodes 9 and 10 in Fig. 1). For the Cyclophoroidea species, there is no established molecular clock rate to estimate the divergence time. Therefore, we estimated the divergence time of the present species using the molecular clock rate (Method C) as proposed for the COI gene for different Protostomia groups based on known vicariance events (ranging from 0.0125 to 0.0206, substitutions per site and Ma, in reference to the substitution models HKY + G)^40^. In addition, the GTR + G and HKY + G model was applied to the H3 and 16S sequences. The uncorrelated lognormal relaxed clock model was used for all analyses. We also set the parameters of the site models to Gamma category count = 4 and shape = 1.0 as described by Heath^59^. The Yule process was used to model speciation. The Monte Carlo Markov chain was run four times for 150 million generations (fossil calibration, Fig. 1a), 330 million generations (fossil calibration, Fig. 1b), and 340 million generations (molecular clock, Fig. S1) with sampling every 1000 generations to ensure that the effective sample size (ESS) values were above 200 for all parameters. The first 10% of trees in each run were discarded as burn-in, and the remaining trees were combined to produce an ultra-metric consensus tree using LogCombiner and TreeAnnotator v1.5.3 (included in the BEAST software package).

## Supplementary information

SUPPLEMENTARY INFO

## Data Availability

All data needed to evaluate the conclusions in the paper are present in the paper and/or the Supplementary Materials. Additional data related to this paper may be requested from the corresponding author. Higher-resolution figures have been deposited in the figshare database (https://doi.org/10.6084/m9.figshare.9973604).

## References

[1] Schluter, D. *The ecology of adaptive radiation* (Oxford University Press, New York, 2000).

[2] Hunt G (2007). The relative importance of directional change, random walks, and stasis in the evolution of fossil lineages. Proc. Natl. Acad. Sci. USA.

[3] Xing L (2016). A feathered dinosaur tail with primitive plumage trapped in mid-Cretaceous amber. Curr. Biol..

[4] Xing L (2017). A mid-Cretaceous enantiornithine (Aves) hatchling preserved in Burmese amber with unusual plumage. Gondwana Res..

[5] Cardinal S, Danforth N (2013). Bees diversified in the age of eudicots. Proc. R. Soc. B.

[6] Vermeij GJ (1977). The Mesozoic marine revolution: Evidence from snails, predators and grazers. Paleobiology.

[7] Payne JL (2005). Evolutionary dynamics of gastropod size across the end-Permian extinction and through the Triassic recovery interval. Paleobiology.

[8] Knight, J. B. *et al*. Systematic Descriptions. in Treatise on Invertebrate Paleontology, Part I, Mollusca 1, R. C. Moore, Eds, pp. I169–I324 (Geological Society of America and University of Kansas Press, Lawrence, 1960).

[9] Emerson WK (1962). A classification of the scaphopod mollusks. J. Paleontol..

[10] Rorsenberg G (1996). Independent evolution of terrestriality in Atlantic truncatellid gastropods. Evolution.

[11] Kano Y, Neusser TP, Fukumori H, Jöger KM, Schrödl M (2015). Sea-slug invasion of the land. Biol. J. Linn. Soc..

[12] Dinapoli A, Klussmann-Kolb A (2010). The long way to diversity–Phylogeny and evolution of the Heterobranchia (Mollusca: Gastropoda). Mol. Phylogenet. Evol..

[13] Dayrat B (2011). Phylogenetic relationships and evolution of pulmonate gastropods (Mollusca): new insights from increased taxon sampling. Mol. Phylogenet. Evol..

[14] Páll-Gergely B, Naggs F, Asami T (2016). Novel shell device for gas exchange in an operculate land snail. Biol. Lett..

[15] Páll-Gergely B, Fehér Z, Hunyadi A, Asami T (2015). Revision of the genus Pseudopomatias and its relatives (Gastropoda: Cyclophoroidea: Pupinidae). Zootaxa.

[16] Raheem CD, Schneider S, Böhme M, Vasiloyan D, Prieto J (2017). The oldest known cyclophoroidean land snails (Caenogastropoda) from Asia. J. Syst. Palaeontol..

[17] Azuma, M. *Colored illustrations of the land snails of Japan*. (Hoikusha, Osaka, 1982).

[18] Yu T, Wang B, Pan H (2018). New terrestrial gastropods from mid-Cretaceous Burmese amber. Cretaceous Res..

[19] Xing L, Ross AJ, Stilwell JD, Fan J, McKellar RC (2019). Juvenile snail with preserved soft tissue in mid-Cretaceous amber from Myanmar suggests a cyclophoroidean (Gastropoda) ancestry. Cretaceous Res..

[20] Neubauer, T. A., Páll-Gergely, B., Jochum, A. & Harzhauser, M. Striking case of convergence—Alleged marine gastropods in Cretaceous Burmese amber are terrestrial cyclophoriods. Comment on Yu *et al*. Palaeoworld, in press (2019).

[21] Rundell RJ (2008). Cryptic diversity, molecular phylogeny and biogeography of the rock- and leaf litter-dwelling land snails of Belau (Republic of Palau, Oceania). Philos. Trans. R. Soc. London, Ser. B.

[22] Webster NB, Van Dooren TJ, Schilthuizen M (2012). Phylogenetic reconstruction and shell evolution of the Diplommatinidae (Gastropoda: Caenogastropoda). Mol. Phylogenet. Evol..

[23] Lee YC, Lue KY, Wu WL (2008). A molecular phylogenetics investigation of Cyathopoma (Prosobranchia: Cyclophoridae) in East Asia. Zool. Stud..

[24] Naggs F, Raheem DC (2005). Sri Lankan snail diversity: faunal origins and future prospects. Rec. West. Aust. Mus. Suppl..

[25] Boonngam P, Dumrongrojwattana P, Matchacheep S (2008). The diversity of land snail fauna in Chonburi Province, Eastern Thailand. Kasetsart J. (Nat. Sci.).

[26] Raheem DC (2014). A Systematic Revision of the Land Snails of the Western Ghats of India. Trop. Nat. Hist., Suppl..

[27] Budha PB, Naggs F, Bacjeljau T (2015). Annotated Checklist of the Terrestrial Gastropods of Nepal. ZooKeys.

[28] Phung C-C, Yu FTY, Liew T-S (2017). A checklist of land snail from the west coast islands of Sabah, Borneo (Mollusca, Gastropoda). ZooKeys.

[29] Shi G (2012). Age constraint on Burmese amber based on U–Pb dating of zircons. Cretaceous Res..

[30] van Benthem Jutting WSS (1948). Critical revision of the Japanese operculate land-shells of the families Hydrocenidae, Helicinidae, Cyclophoridae, Pupinidae and Cochlostomatidae. Treubia.

[31] van Benthem Jutting WSS (1963). Non-marine mollusca of West New Guinea. Part 2, Operculate land shells. Nova Guinea, Zool..

[32] Maassen WJM (1997). A preliminary checklist of the terrestrial molluscs of Sulawesi, Indonesia. A new start? De Kreukel.

[33] Maassen WJM (2001). A oreliminary checklist of the non-marine molluscs of West-Malaysia. A handlist. De Kreukel Suppl..

[34] Yen T-C (1939). Die chinesischen Land- und Süßwasser-Gastropoden des Natur-Museums Senckenberg. Abh. Senckb. Naturforsch. Ges..

[35] Yen T-C (1941). Notes on the genus Lagochilus Blanford, with special reference to its Chinese species. The Nautilus.

[36] Yen T-C (1942). A review of Chinese gastropods in the British Museum. Proc. Malacol. Soc. London.

[37] Beets C (1986). Molluscan fauna of the Lower Gelingseh Beds s. str., Sangkulirang area, Kalimantan timur (East Borneo). Scripta Geol..

[38] Páll-Gergely B, Nguyen PK, Chen Y (2019). A review of Vietnamese Schistoloma Kobelt, 1902 with a list of all known species of the genus (Caenogastropoda: Cyclophoroidea: Pupinidae). Raffles Bull. Zool..

[39] Bandel K (1991). Gastropods from brackish and fresh water of the Jurassic-Cretaceous transition (a systematic reevaluation). Berl. Geowiss. Abh..

[40] Wilke T, Schultheiß R, Albrecht C (2009). As time goes by: a simple fool’s guide to molecular clock approaches in invertebrates. Am. Malacol. Bull..

[41] Kobayashi K (2004). Origin of the Palau and Yap trench-arc systems. Geophys. J. Int..

[42] Schulte P (2010). The Chicxulub Asteroid Impact and Mass Extinction at the Cretaceous-Paleogene Boundary. Science.

[43] Chiba S (1999). Accelerated evolution of land snails Mandarina in the oceanic Bonin Island: evidence from mitochondrial DNA sequences. Evolution.

[44] Noshita K, Asami T, Ubukata T (2012). Functional constraints on coiling geometry and aperture inclination in gastropods. Paleobiology.

[45] Schilthuizen M (2006). Microgeographic evolution of snail shell shape and predator behaviour. Evolution.

[46] Schilthuizen M (2007). Sexual selection maintains whole-body chiral dimorphism in snails. J. Evol. Biol..

[47] Yamamoto S, Takahashi Y, Parker J (2017). Evolutionary stasis in enigmatic jacobsoniid beetles. Gondwana Res..

[48] Wada S, Kameda Y, Chiba S (2013). Long-term stasis and short-term divergence in the phenotypes of microsnails on oceanic islands. Mol. Ecol..

[49] Cai C-Y (2014). Early origin of parental care in Mesozoic carrion beetles. Proc. Natl. Acad. Sci. USA.

[50] Gude, G. K. *The Fauna of British India, Including Ceylon and Burma. Mollusca.−III. Land operculates (Cyclophoridae, Truncatellidae, Assimineidae, Helicinidae)*. (Taylor and Francis, London, 1921).

[51] Goodfriend GA (1986). Variation in land-snail shell form and size and its causes: A review. Syst. Zool..

[52] Kimura K, Hirano T, Chiba S (2015). Assortative mating with respect to size in the simultaneously hermaphroditic land snail Bradybaena pellucida. Acta Ethol..

[53] Poinar GO, Roth B (1991). Terrestrial snails (Gastropoda) in Dominican amber. The Veliger.

[54] Hirano T, Kameda Y, Kimura K, Chiba S (2014). Substantial incongruence among the morphology, taxonomy, and molecular phylogeny of the land snails Aegista, Landouria, Trishoplita, and Pseudobuliminus (Pulmonata: Bradybaenidae) occurring in East Asia. Mol. Phylogenet. Evol..

[55] Grimaldi D, Engel MS, Nascimbene P (2002). Fossiliferous Cretaceous amber from Myanmar (Burma): its rediscovery, biotic diversity, and paleontological significance. Am. Mus. Novit..

[56] Cox, L. R. General characteristics of Gastropoda. In: Treatise on Invertebrate Paleontology, Part I, Mollusca 1, Moore. R. C. ed. (Geological Society of America and University of Kansas Press, Lawrence, 1960), pp. I85–I154.

[57] Arnold WH (1965). A glossary of a thousand-and-one terms used in conchology. The Veliger, Suppl..

[58] Bouckaert R (2014). BEAST2: A software platform for Bayesian evolutionary analysis. PLoS Comput. Biol..

[59] Heath, T. A. Divergence Time Estimation using BEAST v2. Dating Species Divergences with the Fossilized Birth-Death Process. Source, https://taming-the-beast.org/tutorials/FBD-tutorial/FBD-tutorial.pdf, (Tutorial written for workshop on applied phylogenetics and molecular evolution, Bodega Bay, California), pp. 1–44 (2015).

