# Publisher Correction: Cretaceous amber fossils highlight the evolutionary history and morphological conservatism of land snails

**DOI:** 10.1038/s41598-019-55311-7

**Published:** 2019-12-09

**Authors:** Takahiro Hirano, Kaito Asato, Shûhei Yamamoto, Yui Takahashi, Satoshi Chiba

**Affiliations:** 1 Department of Biological Sciences, University of Idaho, Moscow, USA; 2 Faculty of Life and Environmental Sciences, University of Tsukuba, Ibaraki, Japan; 3 Integrative Research Center, Field Museum of Natural History, Chicago, USA; 4 Muroto Geopark Promotion Committee, Muroto Global Geopark Center, Kochi, Japan; 5 Center for Northeast Asian Studies, Tohoku University, Miyagi, Japan; 6 Graduate school of Life Sciences, Tohoku University, Miyagi, Japan

Correction to: *Scientific Reports* 10.1038/s41598-019-51840-3, published online 04 November 2019

The publication date was omitted from the original PDF version of this Article. This error has now been corrected in the PDF version; the HTML version was correct from the time of publication.

Additionally, the URL in the section entitled “Data Availability” did not link to the correct figshare database. This has been corrected in both the HTML and PDF versions.

