# Supplementary material for: Cretaceous amber fossils highlight the evolutionary history and morphological conservatism of land snails
Source: Sci Rep. 2019 Nov 4;9:15886. doi: 10.1038/s41598-019-51840-3 (PMC6828811; doi:10.1038/s41598-019-51840-3)
Supplement: Supplementary file 1 — SUPPLEMENTARY INFO [file 41598_2019_51840_MOESM1_ESM.pdf]

Title: Cretaceous amber fossils highlight the evolutionary history and morphological conservatism of land snails

Authors

Takahiro Hirano[1, *,†], Kaito Asato[2, *], Shûhei Yamamoto[3, *], Yui Takahashi[4] & Satoshi Chiba[5, 6]

Affiliations

[1]Department of Biological Sciences, University of Idaho, Moscow, USA

[2]Faculty of Life and Environmental Sciences, University of Tsukuba, Ibaraki, Japan

[3]Integrative Research Center, Field Museum of Natural History, Chicago, USA

[4]Muroto Geopark Promotion Committee, Muroto Global Geopark Center, Kochi, Japan

[5]Center for Northeast Asian Studies, Tohoku University, Miyagi, Japan

[6]Graduate school of Life Sciences, Tohoku University, Miyagi, Japan

†Corresponding author. Email: hirano0223t@gmail.com (T. H.)

* These authors contributed equally to this work as co-first authors.

Supplementary Materials

Supplementary text

Supplementary figures

Fig. S1. Maximum clade credibility trees generated with the BEAST2 analysis from the combined sequences (COI, 16S, and H3 genes) using a COI molecular clock rate.

Fig. S2. Micro-CT scanning image of *Lagocheilus cretaspira* and *Lagocheilus electrospira* Asato and Hirano, sp. nov. from the Cretaceous Burmese amber.

Fig. S3. Cyclophoridae sp. 1 from the mid-Cretaceous Burmese amber.

Fig. S4. Cyclophoridae sp. 2 from the mid-Cretaceous Burmese amber.

Fig. S5. Cyclophoridae sp. 3 from the mid-Cretaceous Burmese amber.

Fig. S6. Cyclophoridae sp. 4 from the mid-Cretaceous Burmese amber.

Supplementary tables

Supplementary tables in this manuscript longer than one page, hence we provide tables as another word file. Table captions are here:

Table S1. Mesozoic to early Cenozoic fossil records of the Cyclophoridae, Diplommatinidae, and Pupinidae.

Table S2. Detailed results of divergence time estimation of each node in Fig. 1 and Fig. S1 (Lower CI, Upper CI).

Table S3. Taxonomy and distribution of *Schistoloma*[28, 29].

Table S4. Sampling localities and Genbank accession number of each DNA sequences.

Table S5. Information on sequence alignments.

Supplementary video

Supplementary video in this manuscript are provided as H.264 encoding, the aspect ratio of 4:3. Caption is here:

Video S1. Reconstructed movie based on micro-CT scanning of *Schistoloma electrothauma* Asato and Hirano sp. nov. Copyright © 2017 Shimadzu Corporation.

Supplementary Text

Systematic Palaeontology of four fossil specimens

Systematic Palaeontology

Superfamily: **Cyclophoroidea** Gray, 1847

Family: **Cyclophoridae** Gray, 1847

Cyclophoridae sp. 1

Figure 6b, S3

Description: The shell very small and thin, discoidal approximately 1.0 mm high and 3.9 mm wide. Spire whorls curved and inflated with a shallow suture. Apex blunt, and embryonic shell flat, but difficult to see detail of surface of embryonic shell. Aperture ovoid or elliptical, but broken. Excrements around the aperture elliptical. Umbilicus completely open and wide, about one third of diameter of shell. Shell surface of umbilicus having very slightly growth line. Last whorl having spiral ribs.

Locality and Horizon: Burmese amber from Hukawng Valley (26°15′N, 96°34′E), Kachin State, northern Myanmar; lowermost Cenomanian (ca. 99 Ma[1]), Upper Cretaceous.

Material: Data label: NMNS PM 28275. Deposited in National Museum of

Nature and Science, Tsukuba, Ibaraki, Japan (NMNS).

Remarks: Probably soft body is in the shell. Excrements are around the aperture, and its shape is similar to that of extant Cyclophoroidea. Considering shell morphology of the specimen, we treat this specimen as Cyclophoridae. This species can be distinguished from other similar species such as *Archaeocyclotus* gen. nov., Cyclophoridae sp. 2, and Cyclophoridae sp. 3 by spiral ribs. However, this individual seems to not be an adult and its aperture is broken, so we do not identify any genus or species.

Cyclophoridae sp. 2

Figure 6c, S4

Description: Shell very small and thin, crushed the bottom side, and discoidal approximately 1.1 mm high and 2.4 mm wide. Spire whorls curved and inflated with a deep and impressed suture and slightly growth line, and gradually increasing vertical ribs toward aperture. Aperture ovoid or elliptical. Apex blunt, and embryonic slightly sphere-like shape with smooth and round whorls. Excrements around the aperture elliptical.

Locality and Horizon: Burmese amber from Hukawng Valley (26°15′N, 96°34′E), Kachin State, northern Myanmar; lowermost Cenomanian (ca. 99Ma[1]), Upper

Cretaceous.

Material: Data label: NMNS PM 28276. Deposited in NMNS.

Remarks: Excrements are preserved in the amber such as at the above of the aperture, and its shape is similar to that of extant Cyclophoroidea. Considering shell morphology of the specimen, we treat this specimen as Cyclophoridae. This species can be distinguished from other similar species such as Cyclophoridae sp. 1, and Cyclophoridae sp. 3 by vertical ribs. Compared with *Archaeocyclotus* gen. nov., which has also vertical ribs, Cyclophoridae sp. 2 does not have hair-like periostracum. However, this individual seems to not be an adult and the bottom side of the shell is crushed, so we do not identify any genus and species.

Cyclophoridae sp. 3

Figure 6d, S5

Description: Shell very small and thin, crushed bottom side, and discoidal approximately 2.5 mm high and 5.0 mm wide. Spire whorls curved and inflated with a shallow suture, and having growth line. Last whorl around aperture having spiral ribs. Excrements around the aperture elliptical. Apex blunt, and embryonic slightly sphere-like shape with smooth and round whorls.

Locality and Horizon: Burmese amber from Hukawng Valley (26°15′N, 96°34′E), Kachin State, northern Myanmar; lowermost Cenomanian (ca. 99Ma[1]), Upper Cretaceous.

Material: Data label: NMNS PM 28277. Deposited in NMNS.

Remarks: Excrements are preserved in the amber such as at the above of the aperture, and its shape is similar to that of extant Cyclophoroidea. Considering shell morphology of the specimen, we treat this specimen belongs to Cyclophoridae. This species can be distinguished from other similar species such as *Archaeocyclotus* gen. nov., Cyclophoridae sp. 1, and Cyclophoridae sp. 2 by smooth surface of the shell. However, this individual seems to not be an adult and the bottom side of the shell is crushed, so we do not identify any genus or species.

Cyclophoridae sp. 4

Figure 6a, S6

Description: Shell grobosely conical, thin and minute, 2.2 mm in shell length and 2.2 mm in shell width. Whorls 3.3 turns, 1 to 2 whorls smooth and after 2 whorls gradually becoming angulation near middle position of whorls. Base flattened with narrow umbilicus. Aperture roundish rhomboidal, and peristome not continuous. Outer

surface of body whorl sculptured fine, prosocline growth lines. Operculum not calcified (probably chitinized), lamellar and thin; a sinistral and multispiral cord present.

Locality and Horizon: Burmese amber from Hukawng Valley (26°15′N, 96°34′E), Kachin State, northern Myanmar; lowermost Cenomanian (ca. 99Ma[1]), Upper Cretaceous.

Material: Data label: NMNS PM 28278. Deposited in NMNS.

Remarks: The morphological characters of the peristome and small number of whorls suggest that this specimen is very likely to be a juvenile. Although it is difficult to identify the species, we believe this specimen belongs to Cyclophoridae due to a lamellar and thin operculum. This specimen has a grobosely conical shell with a smooth surface, similar to the extinct genus *Palaeocyclophorus* Wenz, 1923 and the extant genus *Leptopoma* L. Pferffer, 1847. In *Leptopoma*, however, the shell is considerably thick and somewhat smooth. Therefore, this specimen is probably related to *Palaeocyclophorus*, but we do not identify any genus or species.

Notes about taxonomic tables

Palaeocyclophorus heliciformis (Matheron, 1832), *P. heberti* Roule, 1884, *P. luneli* (Matheron, 1842), *P. galloprovincialis* (Matheron, 1842), and *P. solarium*

(Matheron, 1842) were assigned to the recent genus *Cyclophorus* Montfort, 1810 by several researchers[2], but these older described species of *Cyclophorus* were reassigned to *Palaeocyclophorus* or additional closely related taxa based on the shell characters[3]. Hence, we assign these five species to *Palaeocyclophorus*. In addition, *Palaeocyclophorus* sp. was also seen in several sections of the French Maastrichtian[3–6], but most of these records are only listed as the occurrence table of fauna or commented in the section of stratigraphic and biotic context. Another fossil material that was not identified at the species level is *Palaeocyclophorus* sp., occurred in the Upper Cretaceous of Spain[3] with a large and round shell with a smooth shell surface. These characters are clearly different from *A. plicatula* Asato and Hirano, gen. et sp. nov.

According to the fossil records of pupinid land snails[7–23], the five genera have been reported from the Santonian of the Upper Cretaceous to the Lower Oligocene[7]. In these species, *Kallomastoma aberrans*, *Ventriculus dolium* and *Cyclomastoma pachygaster* resemble *S. electrothauma* sp. nov. and have a pupiniform shell profile and a thickened peristome. However, in *K. aberrans*, the shell is much bigger (three times in length and width of *S. electrothauma* sp. nov.) and rounder shell profile than that of *S. electrothauma* sp. nov., and the shape of the aperture is clearly different from that of *S. electrothauma* sp. nov. *Kallomastoma aberrans* has a deformed drop-shape relative to

the former and a circular shape relative to *S. electrothauma* sp. nov. *Ventriculus dolium* and *C. pachygaster* have a small shell but a much rounder shell profile. They can be easily discriminated from *S. electrothauma* sp. nov.

Specifics of DNA extraction, PCR, alignment parameters, and model selection

We extracted DNA and performed PCR-based sequencing of a *Pupinella rufa* individual according to a previous study[24]. Alignment of the COI and H3 sequences was straightforward and required no gaps; 16S sequences were aligned using MUSCLE[25]; GBLOCKS v0.91b[26] was used to select regions in the aligned sequences that were confidently aligned for analysis (Table S5). For divergence time estimation, Kakusan4-4.0.2011.05.28[27] was used to select the appropriate models for sequence evolution.

References in Supplementary Text and Tables

1. Shi, G., Grimaldi, D. A., Harlow, G. E., Wang, J., Wang, J., Yang, M., Lei, W., Li, Q. & Li, X. Age constraint on Burmese amber based on U-Pb dating of zircons. *Cretaceous Res.* **37**, 155–163 (2012).
2. Fabre-Taxy, S. Faunes lagunaires et continentals du Crétacé Supérieur de Province III-Le Maestrichtien et le Danien. *Ann. Paléontol.* **45**, 55–124 (1959).

3. Callapez, P., Barroso-Barcenilla, F., Cambra-Moo, O. & Segura, M. Molluscs from the fossil site of" Lo Hueco"(Upper Cretaceous, Cuenca, Spain): Palaeoenvironmental and sequential implications. *Estudios Geol.* **69**, 227–238 (2013).

4. Kerourio, P. Palaeoenvironmental reconstruction of dinosaur nesting sites based on a geochemical approach to eggshells and associated palaeosols (Maastrichtian, Province Basin, France). *Geobios* **20**, 275–281 (1987).

5. Garcia, G., Pincemaille, M., Vianey-Liaud, M., Marandat, B., Lorenz, E., Cheylan, G., Cappetta, H., Michaux, J. & Sudre, J. Découverte du premier squelette presque complet de *Rhabdodon priscus* (Dinosauria, Ornithopoda) du Maastrichtien inférieur de Provence. *C. R. Acad. Sci.*, *Paris*, *Sci. Terre Planètes* **328**, 415–421 (1999).

6. García, G., Duffaud, S., Feist, M., Marandat, B., Tambareau, Y., Villatte, J. & Sigé, B. La Neuve, gisement à plantes, invertébrés et vertébrés du Bégudien (Sénonien supérieur continental) du basin d'Aix-en-Provence. *Geodiversitas* **22**, 325–348 (2000).

7. Stache, G. Die liburnische Stufe und deren Grenzhorizonte. Eine Studie über die Schichtfolgen der cretacisch-eozänen oder protocänen Landbildungsperiode im

Bereiche der Küstenländer von Österreich-Ungarn. *Abh. k. k. geol. Reichsanst.* **13**, 1–170, plate 1–6 (1889).

8. de Boissy, S. A. Description des coquilles fossils du calcaire lacustre de Rilly-la-Montagne: près de Reims. *Mém. Soc. Géol. France*, *2éme Serie* **3**, 265–285 (1848).
9. Wenz, W. Gastropoda. Teil 1: Allgemeiner Teil und Prosobranchia. In O. H. Schindewolf, ed., *Handbuch der Paläozoologie*, (Gebrüder Borntraeger, Berlin, 1938), Band 6, Lieferung 1.
10. Matheron, M. P. Observations sur les terrains tertiaries des Bouches-du-Rhône, et description des coquilles fossils inédites ou peu connues qu'ils renferment. *Ann. Sci. Indust. Midi France* **3**, 39–80 (1832).
11. Roule, L. *Description de quelques coquilles fossils du calcaire lacustre de Rognac (Bouches-du-Rhône)*. Bulletins de la Société Malacologique de France, 1, Paris (1884).
12. Matheron, M. P. Catalogue méthodique et descriptif des corps organisés fossils du département des Bouches-du-Rhône et lieux circonvoisins; précédé d'un mémoire sur les terrains supérieurs au gré bigarré du S. E. de la France. *Répert. travaux Soc. Staist. Marseille* **6**, 81–341 (1842).
13. Tausch, L. Über die Fauna der nicht-marinen Ablagerungen der oberen Kreide des

Csingerthales bei Ajka im Bakony (Veszpremer Comitat, Ungarn) und über einige Conchylien von Aigen bei Salazburg. *Abh. k. k. geol. Reichsanst. Wien* **12**, 1–32 (1886).

14. Sandberger, F. *Die Land- und Süßwasser-Conchylien der Vorwelt*. 1–96, 1870; 97–160, 1871; 161–256, 1872; 257–352, 1873; 353–616, 1874; 617–1000, 1875 (1870–1875).
15. Maillard, G. Monographie des invertébrés du Purbeckien du Jura. *Mém. Soc. paléont. Suisse* **11**, 1–159 (1885).
16. Klebs, R. Gastropoden im Bernstein. *Jb. preuß. geol. Landesanst.* 366–394 (1886).
17. Yu, T., Wang, B. & Pan, H. New terrestrial gastropods from mid-Cretaceous Burmese amber. *Cretaceous Res.* **90**, 254–258 (2018).
18. Oppenheim, P. Neue Binnenschnecken aus dem Vicentiner Eocän. *Zeitschr. d. Deutsch. geol. Ges.* **47**, 57–193 (1895).
19. De Gregorio, A. Description de certains fossils extramarins de l'Eocén Vicentin. *Ann. Géol. Paléontol.* **10**, 1–27 (1892).
20. Bourguignat, J. R. Note sur quelques coquilles fossiles terrestres et fluviatiles: trouvées dans les dépòts de phosphorites du Département de Tarn-et-Garonne. *Mém. Soc. sci. phys. Nat. Toulouse* **1**, 440–446 (1874).

21. Cossmann, A. L. Catalogue Illustré des Coquilles Fossiles de l'Eocène des Environs de Paris, faisant suite aux travaux paléontologiques de G. P. Deshayes. *Ann. Soc. R. Malacol. Belgique I*: **21**, S. 17-186, Taf. 1-8, 1886; II: **22**, S. 3-214, Taf. 1-8, 1887; III: **23**, S. 3-324, Taf. 1-12, 1888; IV: **24**, S. 3-381, Taf. 1-12, 1889; V + Supplement: **26**, S. 3-163, Taf. 1-3, 1891; App. 1: 28, S.3-18, 1893; App. 2: 31, S. 3-94, Taf. 1-3, 1896; App. 3: 36, S. 9-110, Abb. 1-4, Taf. 2-7, 1902; App. 4: 41, S. 186-286, Taf. 5-10, 1907; App. 5: 49,1913-[1886-1913] (1913).

22. Hrubesch, K. Die santone Gosau-Landschneckenfauna von Glanegg bei Salzburg Österreich. *Mitt. Bayer. Staatssamml. Paläont. hist. Geol.* **5**, 83–120 (1965).

23. Wenz, W. Die Landschneckenkalke des Mainzer Beckens und ihre Fauna. II. Paläontologischer Teil. *Jahrb. Nassauis. Vereins Naturk.* **67**, 30–154, plate 4–11 (1914).

24. Hirano, T., Saito, T. & Chiba, S. The phylogeny of freshwater viviparid snails in Japan. *J. Mollusc. Stud.* **81**, 435–441 (2015).

25. Edgar, R. C. MUSCLE: multiple sequence alignment with high accuracy and high throughput. *Nucleic Acids Res.* **32**, 1792–1797 (2004).

26. Castresana, J. Selection of conserved blocks from multiple alignments for their use in phylogenetic analysis. *Mol. Biol. Evol.* **17**, 540–552 (2000).

27. Tanabe, A. S. Kakusan4 and Aminosan: two programs for comparing nonpartitioned, proportional and separate models for combined molecular phylogenetic analyses of multilocus sequence data. *Mol. Ecol. Resour.* **11**, 914–921 (2011).

28. Bartsch, P. The Philippine land shells of the genus *Schistoloma*. *Proc. U.S. Nat. Mus.* **49**, 195–204 (1915).

29. Páll-Gergely, B., Nguyen, P. K. & Chen, Y. A review of Vietnamese Schistoloma Kobelt, 1902 with a list of all known species of the genus (Caenogastropoda: Cyclophoroidea: Pupinidae). *Raffles Bull. Zool.* **67**, 322–327 (2019).

Supplementary Figures

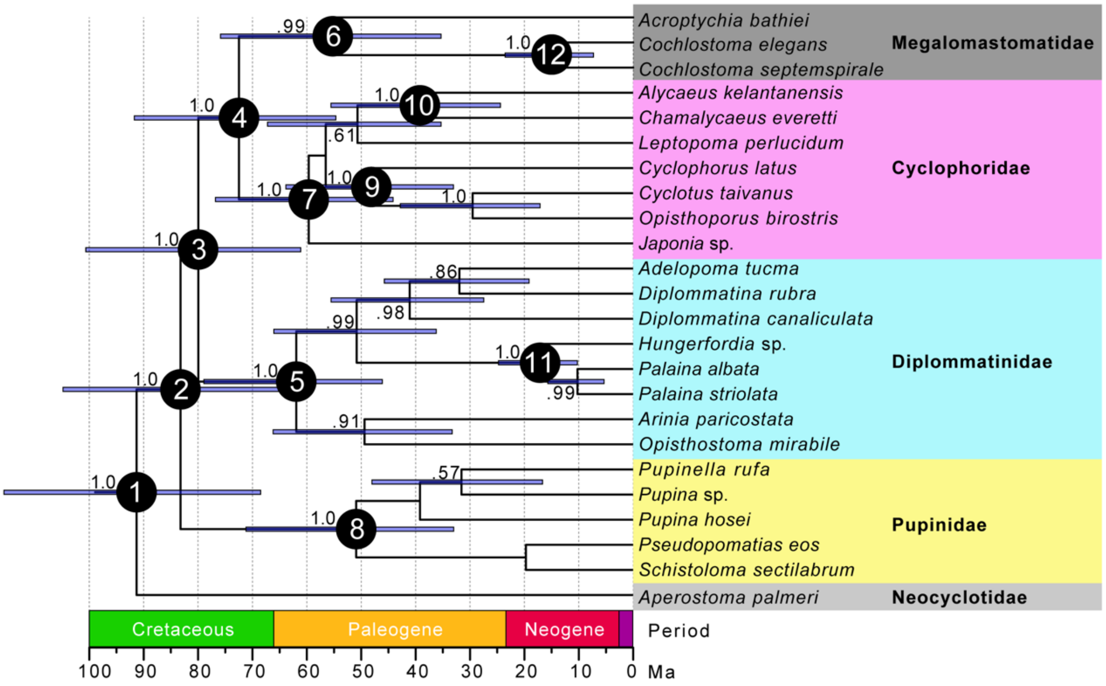

Fig. S1. Maximum clade credibility trees generated with the BEAST2 analysis from the combined sequences (COI, 16S, and H3 genes) using a COI molecular clock rate. The outgroups (*Conus* and *Pomacea*) are not shown. For convenience, we assign numbers to the major nodes (Table S2). Numbers on branches indicate Bayesian posterior probabilities. The node bars indicate a 95% CI for the divergence times. The principal nodes are named by nominal numbers.

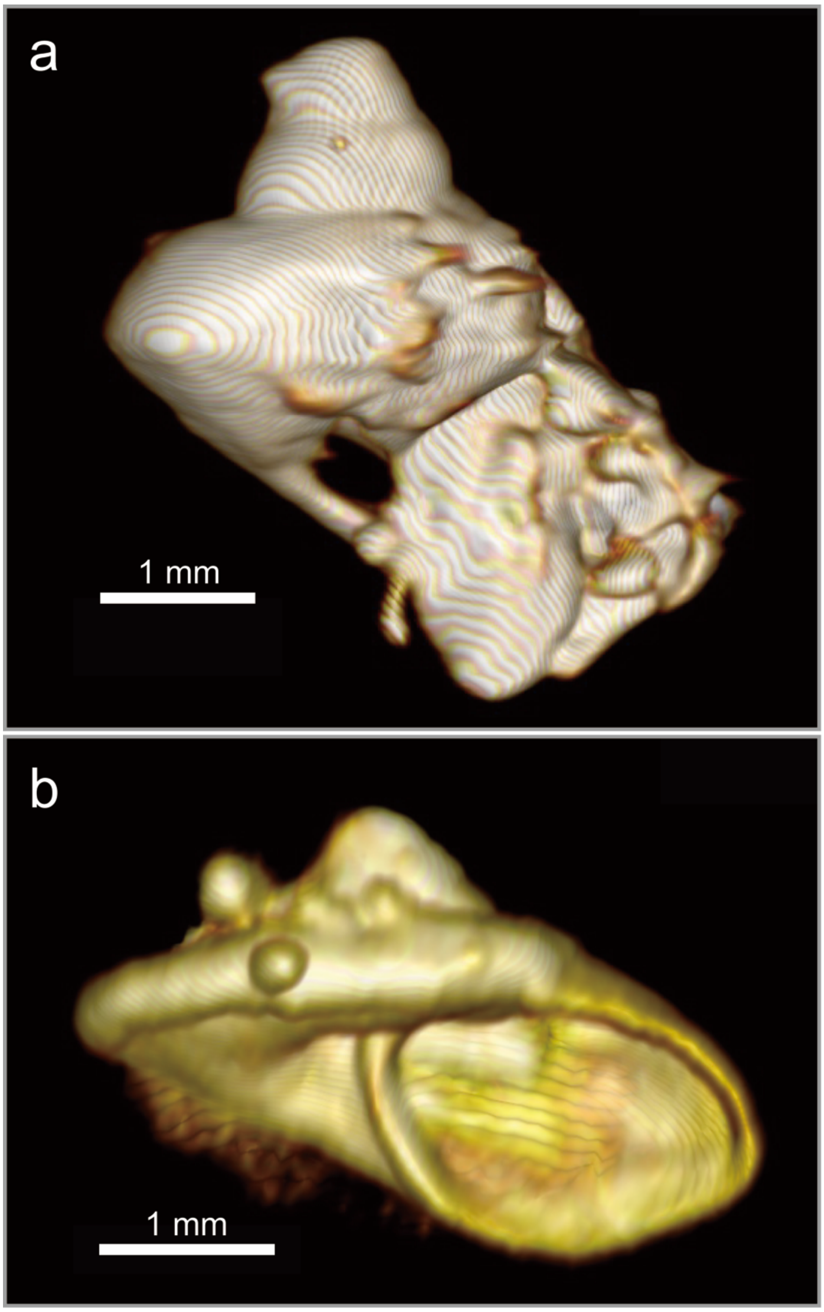

Fig. S2. Micro-CT scanning image of *Lagocheilus cretaspira* Asato and Hirano, sp. nov. and *Lagocheilus electrospira* Asato and Hirano, sp. nov. **a** Holotype of *L. cretaspira* Asato and Hirano, sp. nov. (NMNS PM 28272). **b** Holotype of *L. electrospira* Asato and Hirano, sp. nov. (NMNS PM 28273).

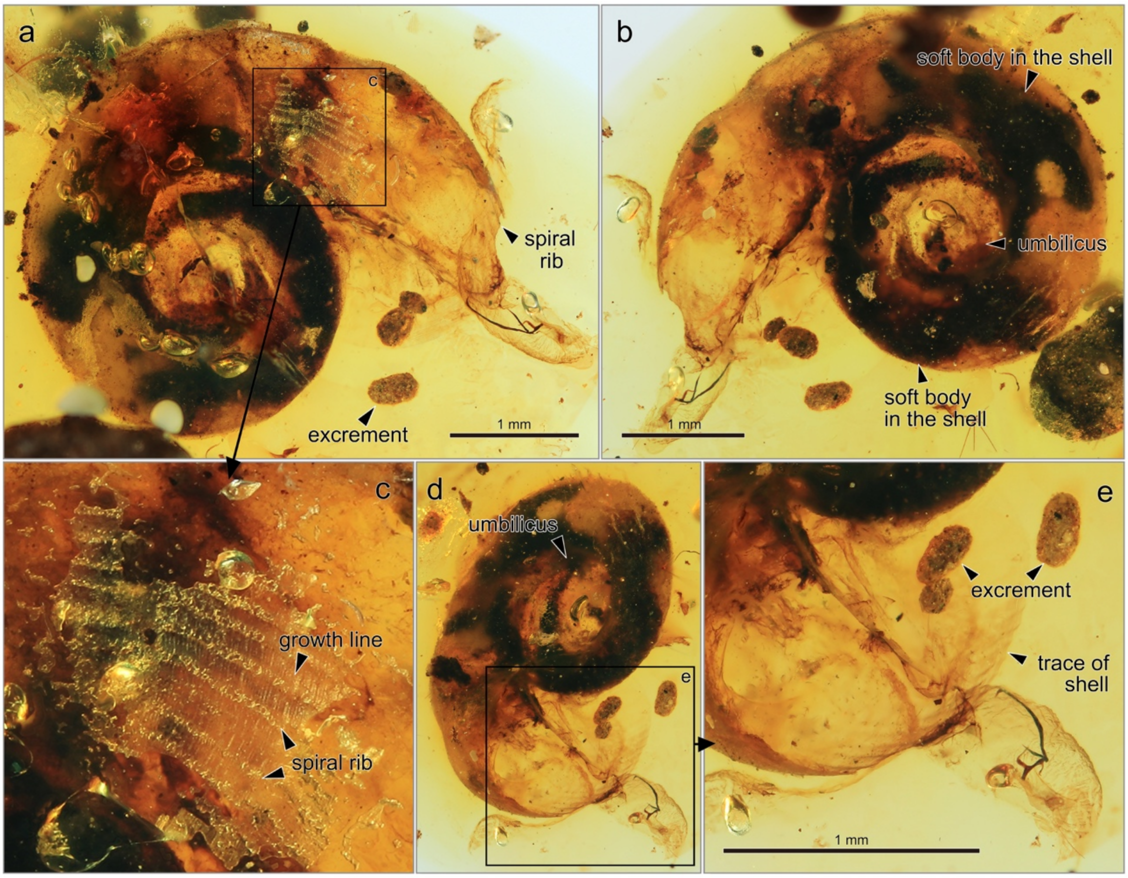

Fig. S3. Cyclophoridae sp. 1 from the mid-Cretaceous Burmese amber. **a**–**e** NMNS PM 28275. **a** Apical view showing spiral ribs and elliptical excrements. **b** Ventral view showing an umbilicus and soft bodies in the shell. **c** Close-up of square **c** in **a** showing spiral ribs and growth lines. **d** Oblique apertural view showing an umbilicus and excrements. **e** Close-up of square **e** in **d** showng three excrements and the trace of shell.

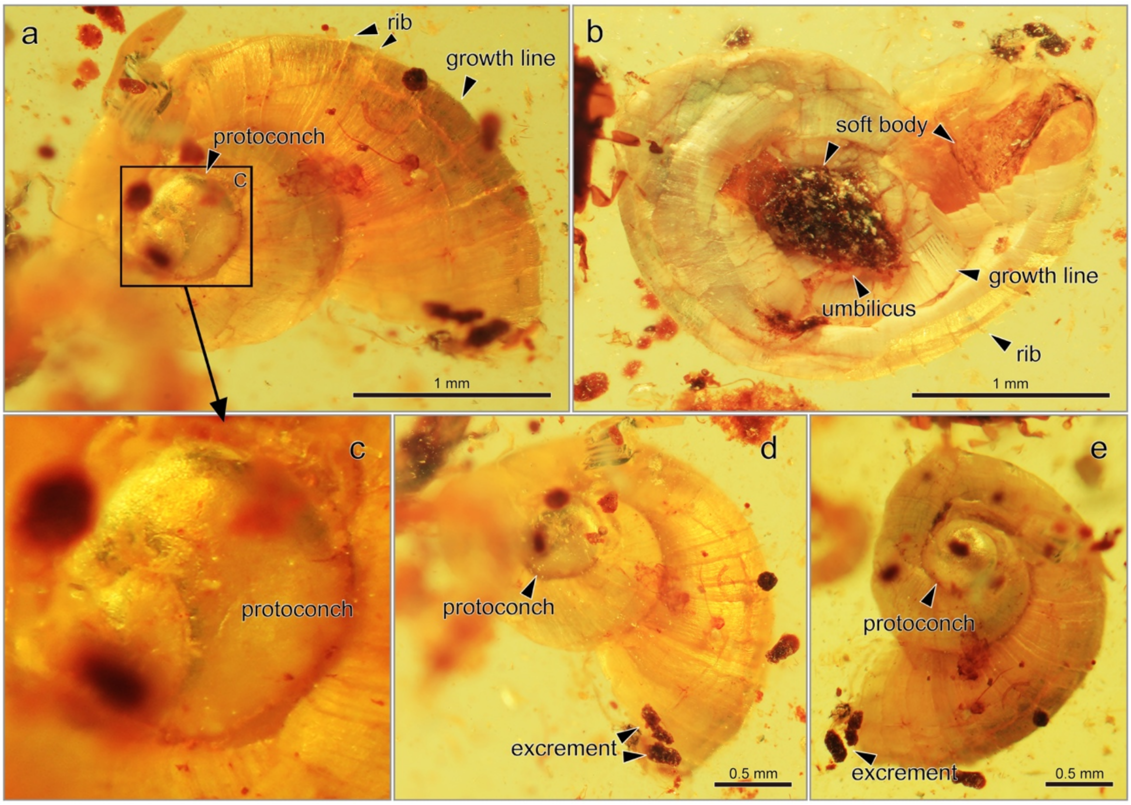

Fig. S4. Cyclophoridae sp. 2 from the mid-Cretaceous Burmese amber. **a**–**e** NMNS PM 28276. **a** Apical view showing a protoconch, growth ribs and lines. **b** Ventral view showing an umbilicus, growth ribs and lines, and soft bodies. **c** Close-up of square **c** in **a** showing a protoconch. **d** Apical view showing a protoconch and excrements. **e** Apical view showing a protoconch and excrements.

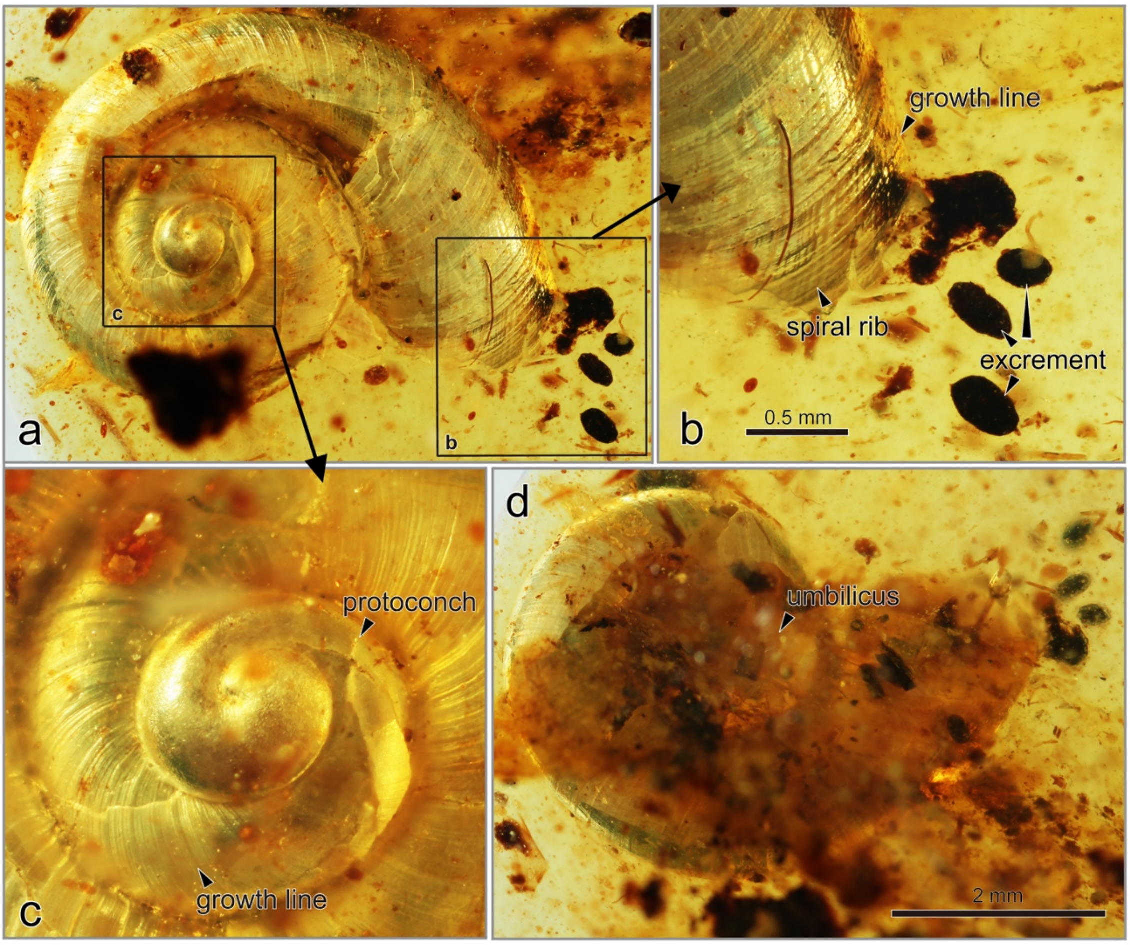

Fig. S5. Cyclophoridae sp. 3 from the mid-Cretaceous Burmese amber. **a**–**d** NMNS PM 28277. **a** Apical view showing a protoconch and excrements. **b** Close-up of square **b** in **a** showing spiral ribs, growth lines and elliptical excrements. **c** Close-up of square **c** in **a** showing a protoconch and growth lines. **d** Ventral view showing an umbilicus.

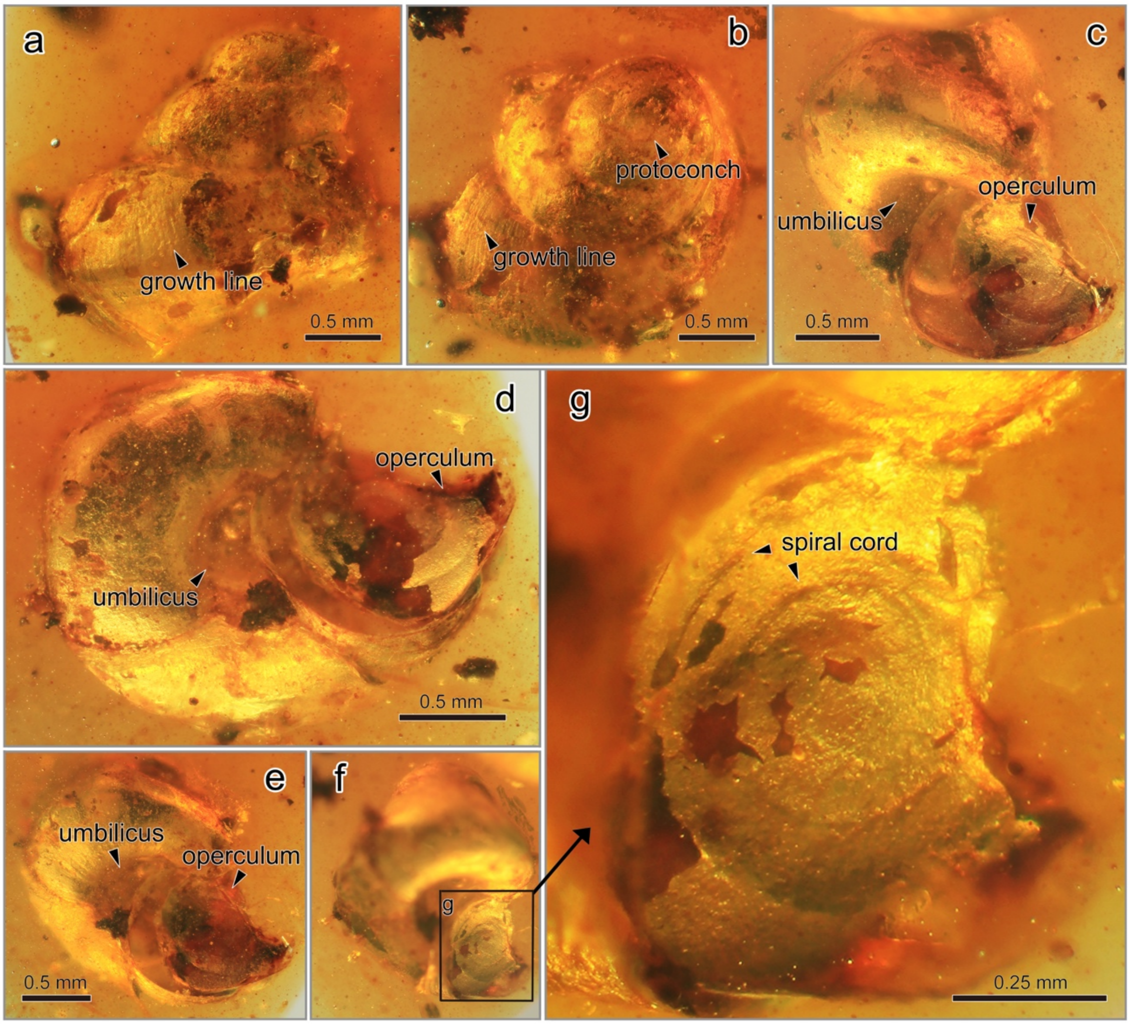

Fig. S6. Cyclophoridae sp. 4 from the mid-Cretaceous Burmese amber. **a–g** NMNS PM 28278. **a** Abapertural view showing growth lines. **b** Apical view showing a protoconch and growth lines. **c** Apertural view showing a narrow umbilicus and an operculum. **d** Oblique apertural view showing an umbilicus and operculum. **e** Oblique apertural view showing an umbilicus and operculum. **f** Oblique apertural view showing an operculum. **g** Close-up of square **g** in **f** showing an operculum with spiral cords.

Table S1. Mesozoic to early Cenozoic fossil records of the Cyclophoridae, Diplommatinidae, and Pupinidae.

Family	Genus	Species	Age	Locality	References
Cyclophoridae Gray, 1847	*Palaeocyclophorus* Wenz, 1923	*P. helicinaeformis* (Boissy, 1848)	Thanetian, Upper Paleocene	Rilly-la-Montagne, France	Boissy (1848), Wenz (1938), Callapez (2013)
		P. heliciformis (Matheron, 1832)	Maastrichtian to Danian, Cretaceous and Lower Paleocene	Les Baux-de-Provence, France	Matheron (1832), Fabre-Taxy (1959)
		P. heberti (Roule, 1884)	Campanian to Danian, Cretaceous and Lower Paleocene	Fuveau, Mimet, Peynier, Puyloubier, Ollieres, Saint-Remy, Les Baux, France	Roule (1884), Fabre-Taxy (1959)
		P. luneli (Matheron, 1842)	Maastrichtian to Danian, Cretaceous and Lower Paleocene	Bouches-du-Rhone, France	Matheron (1832), Fabre-Taxy (1959)
		P. galloprovincialis (Matheron, 1842)	Maastrichtian to Danian, Cretaceous and Lower Paleocene	Ollieres, Saint-Remy, Les Baux, France	Matheron (1842), Fabre-Taxy (1959)
		P. solarium (Matheron, 1842)	Maastrichtian to Danian, Cretaceous and Lower	Rognac, Rousset, Chateauneuf-le-	Matheron (1842), Fabre-Taxy (1959)

			Paleocene	Rouge, Saint-Remy, Organ, Les Baux, France	
		P. sp.	Maastrichtian, Upper Cretaceous	Lo Hueco of Cuenca, Spain	Callapez et al. (2013)
		P. eburneus (Tausch, 1886)	Upper Cretaceous	Ajka in Bakony, Hungary	Tausch (1886)
	"*Cyclotus*" Swainson, 1840	*C. obtusicosta* Sandberger, 1870	Eocene, Paleogene	Pugnello of Veneto region, Italy	Sandberger (1870)
		C. exaratus Sandberger, 1870	Eocene, Paleogene	Pugnello of Veneto region, Italy	Sandberger (1871)
		C. laevigatus Sandberger, 1870	Eocene, Paleogene	Pugnello of Veneto region, Italy	Sandberger (1872)
Pupinidae L. Pfeiffer, 1853	*Ischurostoma* Bourguignat, 1874	*I. filholi* Bourguignat, 1874	Lower Oligocene	Sannois, France	Bourguignat, 1874
		I. formosum formosum (Boubée, 1830)	Lower Oligocene	Sannois, France	Boubée, 1830
		I. imperfectum Stache, 1889	Danian, Paleocene	Istria, Croatia	Stache, 1889
		I. bonneti (Cossman, 1913)	Middle Paleocene	Istria, Croatia	Cossman, 1913
	Rognacia Oppenheim, 1895	*R. abbreviata* (Matheron, 1842)	Maastrichtian to Danian, Cretaceous and Lower Paleocene	Rognac, Rousset, Chateauneuf-le-Rouge, Saint-	Oppenheim, 1895, Matheron, 1842

			Remy, Organ, Les Baux, France	
Kallomastoma Stache, 1889	*K. liburnica* Stache, 1889	Danian, Paleocene	Istria, Croatia	Stache, 1889
	K. compressum Stache, 1889	Danian, Paleocene	Istria, Croatia	Stache, 1889
	K. sublaevigatum Stache, 1889	Danian, Paleocene	Istria, Croatia	Stache, 1889
	K. devestitum Stache, 1889	Danian, Paleocene	Istria, Croatia	Stache, 1889
	K. subimpressum Stache, 1889	Danian, Paleocene	Istria, Croatia	Stache, 1889
	K. tenuitesta Stache, 1889	Danian, Paleocene	Istria, Croatia	Stache, 1889
	K. reductum Stache, 1889	Danian, Paleocene	Istria, Croatia	Stache, 1889
	K. abbreviatum Stache, 1889	Danian, Paleocene	Istria, Croatia	Stache, 1889
	K. inflatum Stache, 1889	Danian, Paleocene	Istria, Croatia	Stache, 1889
	K. impletum Stache, 1889	Danian, Paleocene	Istria, Croatia	Stache, 1889
	K. infranummuliticum Stache, 1875	Danian, Paleocene	Istria, Croatia	Stache, 1889

	K. tergestinum Stache, 1880	Danian, Paleocene	Istria, Croatia	Stache, 1889
	K. distinctum Stache, 1889	Danian, Paleocene	Istria, Croatia	Stache, 1889
	K. strangulatum Stache, 1889	Danian, Paleocene	Istria, Croatia	Stache, 1889
	K. aberrans Stache, 1889	Danian, Paleocene	Istria, Croatia	Stache, 1889
Cyclomastoma Hrubesch, 1965	*C. pachygaster* Hrubesch, 1965	Santonian, Upper Cretaceous	Salzburg, Austria	Hrubesch, 1965
Ventriculus Wenz, 1914	*V. dolium* (Thomae, 1845)	Lower Oligocene	Flörsheim-Hochheim am Main, Germany	Wenz, 1914, Thomae, 1845
Cretatortulosa Yu et al., 2018	*C. multilinea* Yu et al., 2018	Cenomanian, middle Cretaceous	Noije Bum Village of Kachin State, northern Myanmar	Yu et al. (2018)
Pseudopomatias Möllendorff, 1885	*P*? *lyui* Yu et al., 2018	Cenomanian, middle Cretaceous	Noije Bum Village of Kachin State, northern Myanmar	Yu et al. (2019), Neubauer et al. (2019)

The references are in Supplementary Text.

Table S2. Detailed results of divergence time estimation of each node in Fig. 1 and Fig. S1 (Lower CI, Upper CI).

	Fossil calibration		Molecular clock rate (C)
Node	Fig. 1A	Fig. 1B	Fig. S1
1	124.15 (105.63, 146.66)	151.82 (118.14, 192.97)	91.31 (68.50, 115.68)
2	111.73 (100.76, 126.25)	134.92 (109.88, 165.54)	83.24 (63.44, 104.84)
3	106.86 (98.95, 118.27)	127.83 (105.96, 155.56)	79.96 (61.12, 100.63)
4	-	115.56 (100.46, 136.30)	72.48 (54.66, 91.73)
5	77.32 (54.33, 98.63)	94.04 (61.21, 126.48)	61.95 (46.11, 78.93)
6	70.58 (41.61, 95.98)	80.84 (39.57, 117.03)	55.04 (35.33, 75.87)
7	56.40 (33.6, 83.02)	-	59.66 (44.15, 76.80)
8	63.03 (33.69, 95.43)	-	50.93 (33.00, 71.19)
9	-	-	48.14 (33.05, 63.84)
10	-	-	39.50 (24.42, 55.57)
11	22.21 (9.81, 37.33)	28.70 (10.72, 50.29)	17.09 (10.27, 24.77)
12	19.42 (5.37, 37.26)	24.38 (5.27, 49.53)	14.88 (7.3, 23.55)

Table S3. Taxonomy and distribution of *Schistoloma*[28, 29].

Genus	Species	Distribution
Schistoloma Kobelt, 1902	*S. anostoma* (Benson, 1852)	Borneo
	S. doriae (Issel, 1874)	
	S. leferi (Morelet, 1861)	
	S. altum (Sowerby, 1842)	Philippine
	S. mcgregori (Bartsch, 1909)	
	S. quadrasi (Hidalgo, 1889)	
	S. funiculatum (Benson, 1838)	Himalaya
	S. pauperculum (Sowerby, 1843)	
	S. tanychilus (Godwin-Austen, 1876)	Assam
	S. sectilabrum (A. Gould, 1844)	Myanmar<br>peninsular<br>Malaysia<br>Thailand<br>Vietnam
	S. sumatranum (Dohrn, 1881)	Sumatra
	S. cochinchinense (Rochebrune, 1882)	Vietnam
	S. inermis (Bavay & Dautzenberg, 1909)	
	S. maydelineae Páll-Gergely et al., 2019	
	S. messageri (Bavay & Dautzenberg, 1909)	
	S. longyanensis Zhou et al., 2009	China

Table S4. Sampling localities and Genbank accession number of each DNA sequences.

Taxa	No	Sampling site	COI	16S	H3
**Ingroup (GenBank)**					
Acroptychia bathiei	MOL.119775	Madagascar, Ambonarabe	HM753335	HM753491	-
Alycaeus cf. *kelantanensis*	MOL.119765	Malaysia, Perak	-	HM753481	HM753273
Adelopoma tucma	MOL.119828	Argentina, Tucuman	HM753341	HM753534	-
Chamalycaeus everetti	MOL.119764	Malaysia, Borneo, Sabah	HM753329	HM753480	HM753272
Cyclophorus latus	MOL.119768	Taiwan	HM753331	HM753484	HM753275
Cyclotus taivanus	041003-13 (COI), MOL.119769 (16S, H3)	Taiwan	JF913326	HM753485	HM753276
Japonia sp.	MOL.119770	Malaysia, Borneo, Sabah	HM753332	HM753486	HM753277
Leptopoma pellucida	PCC065 (COI, 16S), MOL.119771 (H3)	probably Malaysia (PCC065), Australia, QLD (MOL.119771)	KU986305	KU905048	HM753278
Opisthoporus birostris	MOL.119772	Malaysia, Borneo, Sabah	HM753333	HM753488	HM753279
Cochlostoma elegans	MOL.119773	Croatia, Velebit	HM753334	HM753489	HM753280
Cochlostoma septemspirale	MOL.119825	Switzerland	HM753326	HM753497	HM753269

Arinia paricostata	MOL.119779	Malaysia, Borneo, Sabah	-	HM753500	HM753284
Diplommatina canaliculata	MOL.119783	Malaysia, Pahang	HM753338	HM753504	HM753288
Diplommatina rubra	MOL.119814	Malaysia, Borneo, Sabah	HM753358	HM753516	HM753315
Hungerfordia sp.	MOL.119804	Belau	HM753352	HM753527	HM753306
Opisthostoma mirabile	MOL.119807	Malaysia, Borneo, Sabah	HM753353	HM753529	HM753309
Palaina albata	MOL.119810	Belau, Peleliu	HM753354	HM753531	HM753312
Palaina striolata	MOL.119812	Belau, Ngerekebesang	HM753356	HM753533	HM753313
Aperostoma palmeri	MCZ		DQ093523	DQ093479	DQ093505
Pupina hosei	MOL.119777	Malaysia, Borneo, Sabah	-	HM753493	HM753282
Pupina sp.	FLMNH494200	probably Myanmar	MF983668	-	-
Pseudopomatias eos	P-eos108	Taiwan	-	KP271244	KP271245
Schistoloma sectilabrum	FLMNH494096	probably Myanmar	MF983671	-	-
**Ingroup (This study)**					
Pupinella rufa	HC7215	Japan, Kagoshima, Yakushima	LC500095	LC500096	-

**Outgroup**				
Conus miles		AY588202	FJ868145	AF033684
Pomacea insularum	BR245 (16S, H3), SC_02 (H3)	FJ946828	FJ710229	FJ710375

Table S5. Information on sequence alignments.

Alignment	Length of alignment	Excluded sites
COI	564	-
16S	484	75, 116, 122-124, 162-169, 198-226, 264-301, 309-313, 318-324, 335, 389, 421-438, 453-484
H3	255	-
